# Recent progress of nanomaterials for diagnosis and treatment of rejection in heart transplantation

**DOI:** 10.3389/fbioe.2025.1703902

**Published:** 2025-11-06

**Authors:** Guangyin Li, Chun Wang, Xin Ai, Yuxi Yang, Haizhuo Yu, Piao Wu, Weiyi Zhao, Jiawei Tian, Shuangquan Jiang

**Affiliations:** 1 Department of Ultrasound, The Second Affiliated Hospital of Harbin Medical University, Harbin, China; 2 Ultrasound molecular imaging Joint laboratory of Heilongjiang Province (International Cooperation), Harbin, China

**Keywords:** heart transplantation, acute rejection, nanomaterial, drug delivery, nanotheranostics

## Abstract

Transplant rejection and the side effects of immunosuppressive therapy have hindered heart transplantation development. Rejection of a heart transplant can lead to cellular and antibody-mediated immunoinflammatory responses and allograft dysfunction, thereby significantly affecting patients’ survival and prognosis. To address these challenges, many new technologies and materials, including nanomaterials, have been developed for potential applications in the heart transplantation field. Nanomaterials are most commonly used as drug delivery carriers, and the addition of specific ligands can enhance drug utilization, strengthen therapeutic effects, and reduce the occurrence of adverse reactions. In addition, nanomaterials have been developed as targeted molecular probes to support various imaging techniques and to assist in monitoring the infiltration of immune cells (such as T cells and macrophages) into cardiac tissue, thus facilitating the early diagnosis of acute rejection (AR). Continuous advances in nanotechnology have led to the development of “theranostic” and intelligent-response nanomaterials for precise disease diagnosis and simultaneous treatment. Nanomedicine primarily relies on the development of Nanomaterials and nanostructured surfaces, along with the application of nanotechnology, for molecular diagnosis, therapy, monitoring, and disease treatment. In this review, we examine the recent development of nanomaterials for the diagnosis and treatment of AR in heart transplantation, and discuss the challenges and future directions for the clinical translation of nanomaterials in heart transplantation.

## Graphical Abstract

**Figure:**
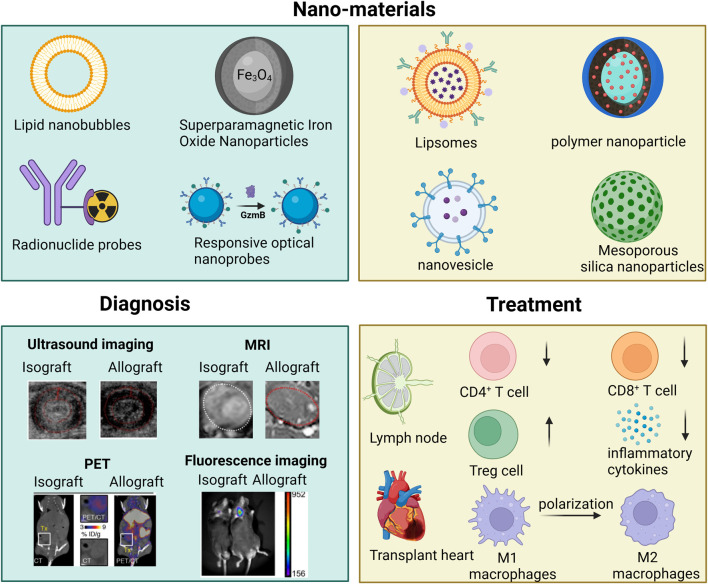
Nanomaterials for diagnosis and treatment of rejection in heart transplantation (Guo et al., 2012; Wu W et al., 2013; Braza et al., 2018; Xu et al., 2020). Created in BioRender.

## 1 Introduction

Heart transplantation is the most effective method for saving the lives of patients with end-stage heart failure. Currently, over 8,000 heart transplants are performed each year across the world (Martin et al., 2025). Although this figure is encouraging, heart transplantation remains highly challenging due to transplantation rejection, individual variations in immunosuppressive therapy, the shortage of donors, and the need for long-term prevention and treatment of graft vascular diseases. Among these challenges, transplant rejection remains the main obstacle limiting the long-term survival of patients (Khush et al., 2021). AR is the most common type of allograft rejection, significantly contributing to morbidity and mortality during the first year following surgery, and approximately 40% of patients experience AR episode within 2 weeks to 3 months after transplantation (Chambers et al., 2021). Responses to heart transplant rejection depend not only on a highly effective diagnosis, particularly in the early stages of rejection, but also on precise and effective treatment.

Rejection involves a series of complex immune processes, and although most researchers have focused on the adaptive immune system, an expanding body of evidence indicates that the innate immune systems’ significant role in rejection (Ochando et al., 2019). Ischemia-reperfusion injury (IRI) is an unavoidable consequence of organ storage and harvesting that releases damage-associated molecular patterns (DAMPs), which are recognized by innate immune cells through pattern recognition receptors (PRRs) (Troise et al., 2025). Activated innate immune cells release proinflammatory cytokines, triggering the complement system and natural killer cells and promoting rejection reactions (Silvis et al., 2020; Li and Lan, 2023). The activation of T cells (the primary immune cells in the adaptive immune system) by innate immune cells, such as antigen-presenting cells (including macrophages, monocytes, and dendritic cells), crucially contributes to allograft rejection (van den Bosch et al., 2017). T cells by directly recognizing alloantigens in donor tissues, can initiate the rejection process. Activated T cells rapidly proliferate and differentiate into effector T cells, which initiate inflammatory responses by secreting various cytokines—such as interferon and tumor necrosis factor—and recruiting other immune cells to participate in the rejection process, thereby exacerbating tissue damage and organ dysfunction (Duneton et al., 2022). In addition, T cells can promote the production of antibodies by B cells or form antigen-specific memory T cells, thereby prolonging the duration of rejection and increasing the risk of repeated rejection (Becker et al., 2021). Current treatments focus on adaptive immunity, while approaches that address IRI and innate immune activation are limited.

**FIGURE 1:**
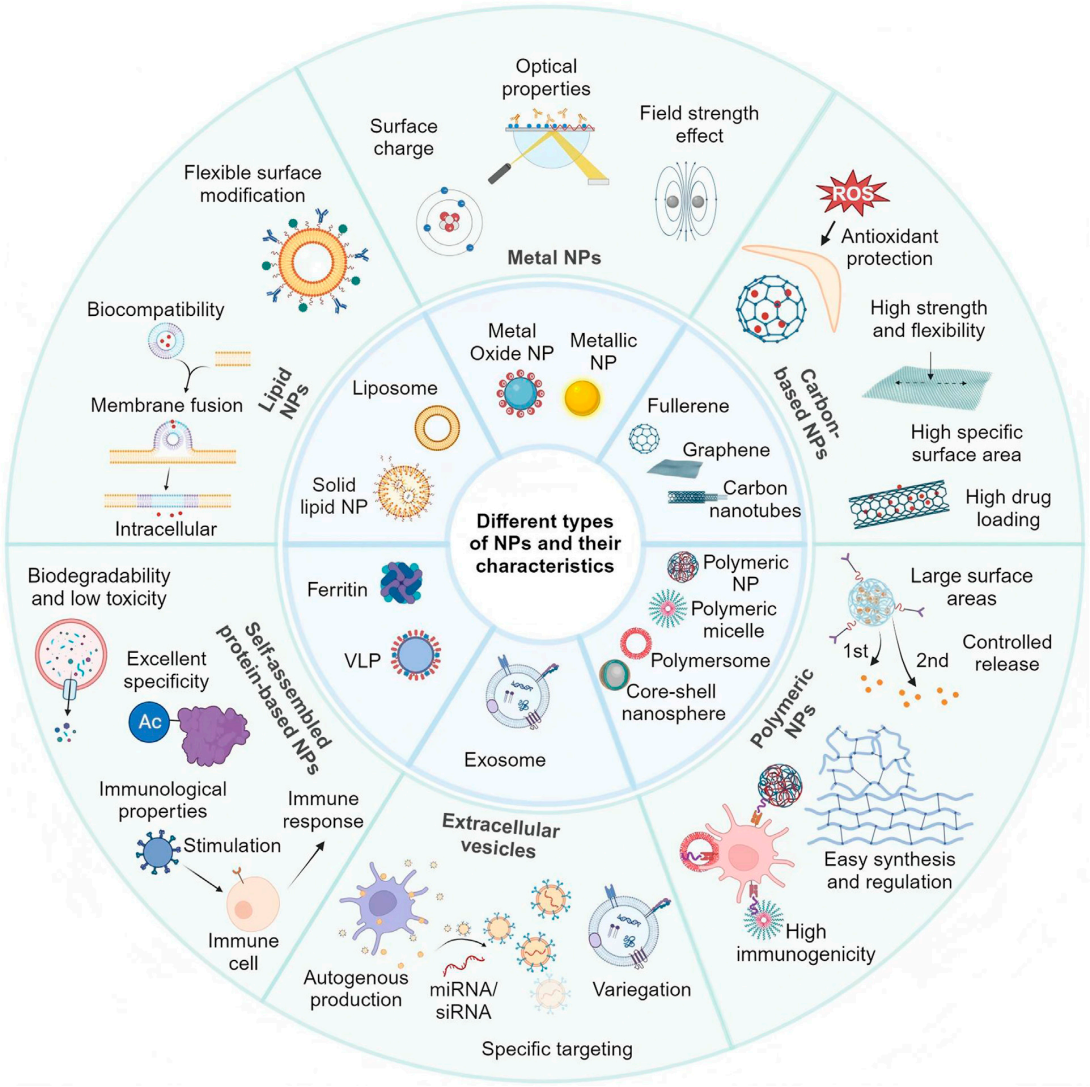
Characteristics of six common nanomaterials. Lipid NPs, composed of lipids like phospholipids, offer excellent biocompatibility and flexible surface modification capabilities. Metal NPs, including metals such as gold, silver, and copper, as well as their oxides, exhibit outstanding optical, electronic, and magnetic properties, making them ideal for biological imaging, photothermal therapy (PTT), and sensing applications. Carbon-based nanomaterials, such as CNTs, graphene, and fullerenes, feature a large surface area, high drug-loading capacity, and chemical stability, providing resistance to oxidative environments. Polymeric NPs, made from various polymers, display diverse structures and properties suitable for multiple biomedical applications. Self-assembled NPs, including ferritin family proteins and virus-like particles (VLPs), offer good biodegradability in the case of ferritin proteins and the ability to mimic viral stimuli to trigger immune responses in the case of VLPs. Exosomes, small vesicles secreted by cells, are rich in proteins, nucleic acids, and signaling molecules, playing vital roles in cellular communication and regulation. These nanomaterials have broad applications in drug delivery, molecular imaging, biosensing, tissue engineering, and disease diagnosis. Adapted with permission from Shen et al. (Huang et al., 2024).

At present, the clinical treatment of heart transplant rejection relies on immunosuppressive medications. Owing to advances in immunosuppressive therapy, the median survival time of transplanted hearts worldwide has increased significantly. However, the long-term use of immunosuppressive drugs and total immunosuppression can result in side effects, including a heightened risk of infection and malignant tumors (Messner et al., 2019). In recent years, with developments in nanotechnology, multiple nanomaterial-based drug delivery systems have been shown to have high efficacy and the potential to achieve targeted drug delivery (Smith, 2021; Smith and Edelman, 2023; Mao et al., 2025). Nanomaterials can be classified based on their chemical compositions as organic-based, inorganic-based, carbon-based, and composite-based nanomaterials, or more specifically into categories such as liposomal nanoparticles, polymeric nanoparticles, protein-based nanoparticles, carbon-based nanoparticles, metal nanoparticles, and extracellular vesicles, etc. (Figure 1). Barhoum et al. (2022), Harish et al. (2022) liposomal nanomaterials have become a benchmark technology in the field of medical nanomaterials over several decades of clinical application due to their advantages, such as efficient drug delivery, precise targeting, innate biocompatibility, and biodegradability (Wahab et al., 2024). The multitude of classifications reflect the abundance of nanotechnology materials, which are expected to play significant roles in the diagnosis and treatment of cardiovascular diseases (Table 1).

**TABLE 1:** Characteristics of common nanomaterials.

Types of NPs	Composition	Typical shape	Surface charge	Efficacy	Safety
Lipid NPs	Liposomes, lecithin, triglycerides, triglycerides of palm stearate, and fatty acids	Sphericl vesicular	Adjustable	High biocompatibility; strong drug loading capacity; flexible surface modification	High safety and can be metabolized into natural lipid components
Metal NPs	Metal and metal oxide NPs,	Pherical, rod-shaped, star-shaped, cubic, etc.	Adjustable	Unique shape, size, structure, and local-field enhancement action	Low safety, difficult to degrade and may remain for a long time
Polymeric NPs	Natural hydrophilic polymers and synthetic hydrophobic polymers	Pherical, micelles, vesicles, irregular shapes, etc.	Depending on the polymer monomer, adjustable	Natural hydrophilic polymers and synthetic hydrophobic polymers	Adjustable, degradable (PLGA), non-degradable (polystyrene)
Protein NPs	VLP proteins	Depending on the protein itself (spherical, cage-like, rod-like, etc.	Depending on the isoelectric point of the protein	Good biocompatibility and biodegradability; multifunctionality through surface modification	High safety, completely degraded into amino acids and safely absorbed and utilized by the body
Exosomes	Classification according to source	Cup-shaped and spherical, with high heterogeneity, etc.	Usually negatively charged	Excellent biocompatibility; targetability	High safety, natural biodegradation pathway
Carbon-based NPs	Carbon nanotubes, Graphene, Fullerenes	Tubular (nanotubes), spherical (fullerenes), sheet-like (graphene)	Depending on the modification	Good chemical and physical stability, Excellent mechanical properties; high strength	Low safety, poor biodegradability and poses a risk of long-term retention

Due to their nanoscale size, nanoparticles exhibit unique biological and physicochemical advantages, such as their ability to pass through cell membranes and tissue barriers. This characteristic enables them to interact with cellular structures of similar dimensions, including proteins and other macromolecules within cells (Zhang et al., 2017). Numerous studies have demonstrated that nanoparticles can significantly enhance targeted accumulation and improve the chemical stability of loaded diagnostic and therapeutic agents, including small molecule drugs, peptides, proteins, small interfering RNAs (siRNAs), and microRNAs (miRNAs) (Kim et al., 2018; Fang et al., 2022). In addition, nanoparticles can be conjugated with bioactive molecules, including cell membranes, specific antibodies, enzymes, and even nucleic acids. These functionalized nanoparticles can bypass immune cells, remain in the body for extended periods, and reach target tissues at higher concentrations by interacting with receptors on the target cells, thus optimizing therapeutic efficiency while minimizing side effects (Liu et al., 2017b; Huang et al., 2023).

In addition to being used as drug carriers, nanoparticles are also being developed as molecular probes for molecular imaging. Molecular imaging involves using noninvasive techniques to visualize and quantitatively assess molecular and cellular biological processes in the body for the prediction, diagnosis, and monitoring of diseases (Grabner et al., 2016; Li et al., 2021). Nanoparticles also have the ability to specifically target cells and enhance imaging sensitivity through surface modification (Deng et al., 2022). Changing the size and surface potential of nanoparticles can reduce additional uptake by the kidneys and liver, allowing the cycle time to be extended, which is conducive to obtaining clearer images (Chen et al., 2023). Finally, nanoparticles can achieve specific controlled releases by incorporating stimulus-responsive functional groups, thereby effectively enhancing image contrast (Ma et al., 2023). Researchers primarily focus on noninvasive imaging of the major immune cells, such as the macrophages and T cells involved in rejection to detect early signs of AR, facilitate timely and effective treatment, and ultimately save patients’ lives.

Nanomaterials, through their unique size effects, functional programmability, and high targeting capabilities, can facilitate more precise and efficient postoperative management of heart transplantation. They are expected to revolutionize the monitoring and treatment of immune rejection, reduce drug side effects, extend the survival time of transplanted hearts, and ultimately improve recipients’ quality of life and long-term survival rates. Although most applications of nanoparticles are still in the preclinical research stage, their potential for future development is widely regarded as a key breakthrough in the field of organ transplantation.

This paper reviews recent advances in diagnostic imaging and drug delivery methods based on nanotechnology in heart transplant rejection, as shown in graphical abstract. While nanomaterials present significant advantages, certain limitations and challenges persist, necessitating continuous research and development. In addition, this paper discusses the prospects, opportunities, and challenges of nanomaterials in heart transplantation, and provides suggestions for future research directions.

## 2 Search strategy

This review followed the PRISMA guidelines. The primary search for article used in this review was conducted using PubMed (168), Web of Science (212), and Science direct (178), and the medical subject headings (heart transplantation, graft rejection, nanoparticles). Using the PubMed database as an example, we present our search strategy: ((“Heart Transplantation*” OR “cardiac transplant*” OR “Heart Transplant*” OR “Heart Graft*” OR “Cardiac Graft*” OR “Transplanted Heart”) OR (“Heart Transplantation” [Mesh])) AND ((Reject* OR “Allograft Reject*” OR “Acute Cellular Rejection” OR “Humoral Reject*” OR “Chronic Reject*” OR “Acute rejectoin”) OR (“Graft Rejection” [Mesh])) AND ((Nano* OR Nanotechnology OR Liposome* OR Micelle* OR Dendrimer* OR “Quantum Dot*” OR “Gold Nanoparticle*” OR SPION OR Exosome* OR “Extracellular Vesicle*” OR Nanocarrier* OR Nanosensor* OR Nanomedicine* OR Nanomaterials*) OR (“Nanostructures” [Mesh])). There is no time limit for article search to identify all published articles.

**FIGURE 2:**
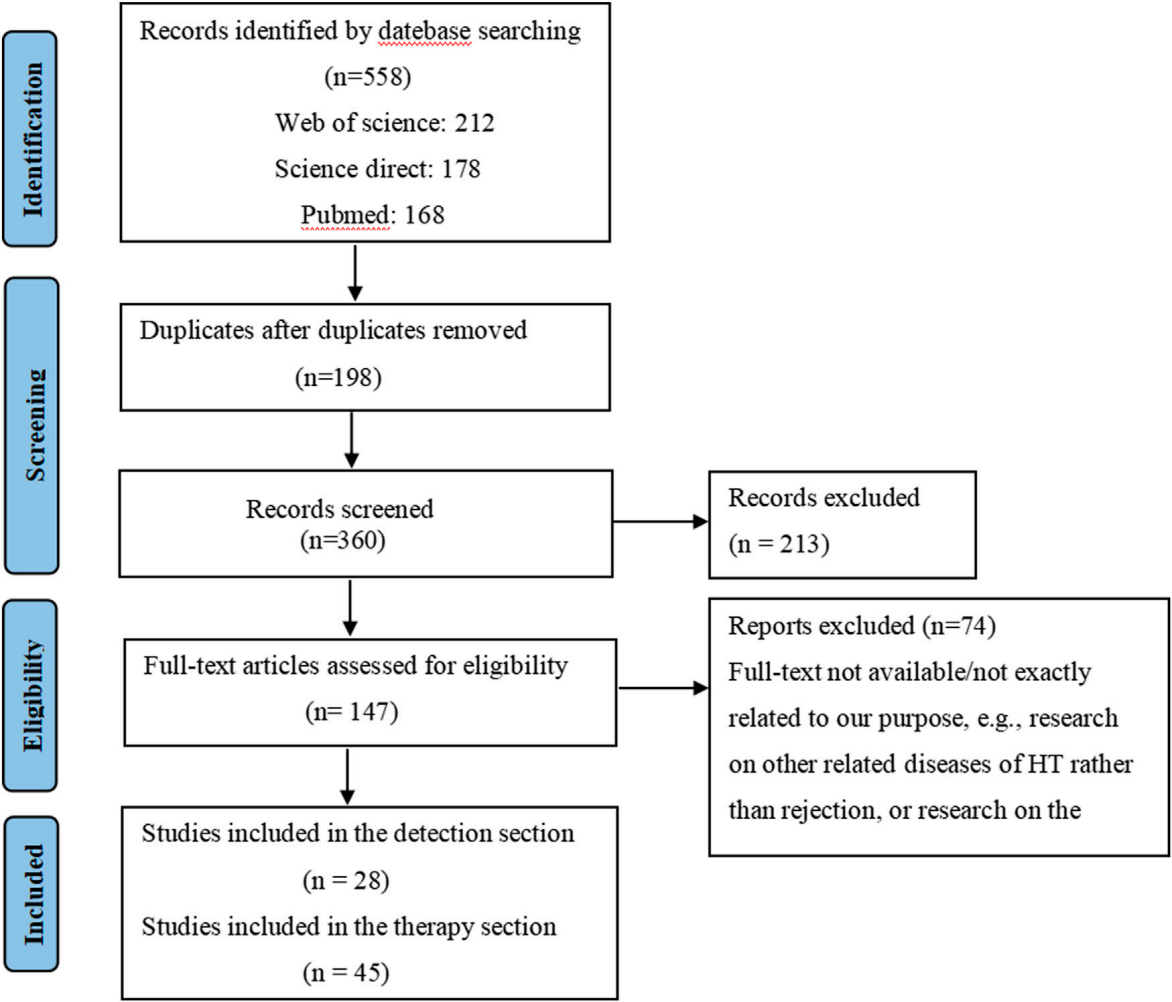
PRISMA flowchart showing the process of article selection.

First, the studies were imported into EndNote X9 (Thomson Reuters, New York, NY, USA) and the duplicates and review articles were removed. Two review authors independently assessed the title and the abstract content (or both) of every record retrieved to decide which studies should be further evaluated and extracted all data. Disagreements were resolved through consensus or by consultation with a third author. Studies were included if they met the following criteria: (1) were written in English with the full text available; (2) used cardiac transplant rejection as the objective disease; (3) were studied *in vitro* by using cellular lines and/or *in vivo* by using animal models; (4) research papers offered at least one treatment plan and/or one detection plan in comparison to routine approaches. Following a rigorous selection process, 83 articles were included in our study. Among these, 28 articles matched the inclusion criteria for the diagnostic section (n = 28) and 45 articles for the treatment section (n = 45). The PRISMA flowchart illustrates the identification and screening process of the articles (Figure 2).

## 3 Nanotechnology for the diagnosis of heart transplant rejection

**FIGURE 3:**
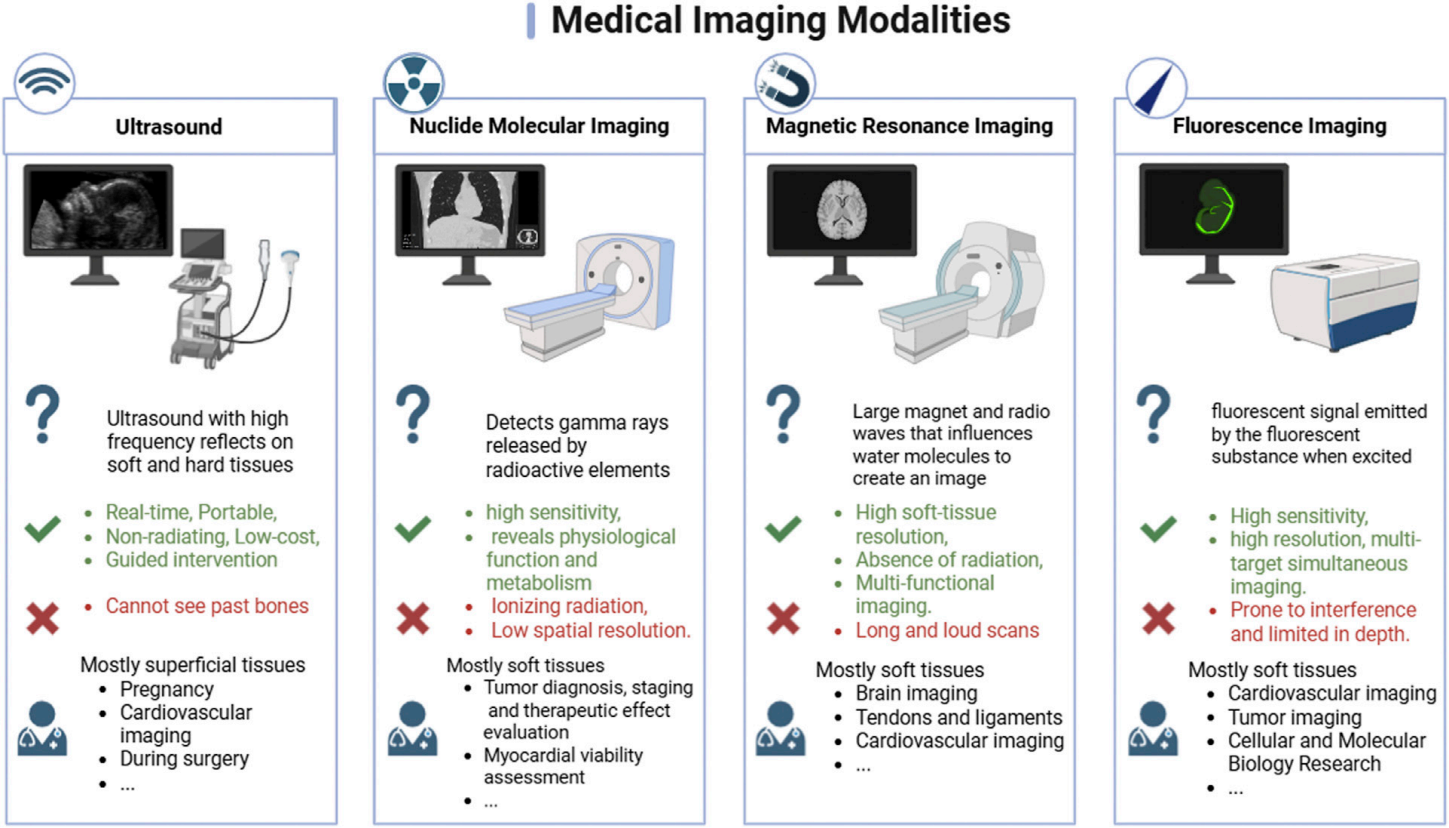
Comparison of common medical imaging modalities: ultrasound, nuclide molecular imaging, magnetic resonance imaging, fluorescence imaging. Created in BioRender.

Real-time monitoring of the immune response is crucial for extending graft survival after transplantation. Endomyocardial biopsy is considered the “gold standard” for diagnosing cardiac rejection and monitoring immunosuppressive therapy. However, this method is invasive and can yield false-negative results. In addition, the stress associated with an invasive endocardial biopsy may further activate the immune response and exacerbate AR (Velleca et al., 2023). Traditional imaging techniques such as computed tomography (CT), magnetic resonance imaging (MRI), and positron emission tomography (PET) have achieved significant success in the diagnosis of various diseases (Figure 3). Unfortunately, reliable imaging tools for the early detection of AR remain scarce to date. Researchers have been striving to develop a sensitive, noninvasive method for diagnosing transplant rejection—for example, genome-based screening for RNA and cell-free DNA analysis, noninvasive detection of circulating or graft-derived exosomes and related proteins. However, nucleic acids and proteins are unstable in circulation, so the reliability of these methods remains questionable (Di Francesco et al., 2018; Castellani et al., 2020).

A variety of nanoprobes have been developed for different imaging modalities and are tailored to meet specific imaging requirements. For example, magnetic iron oxide nanoparticles and nanoparticles modified with signaling elements—such as fluorescent pigments and radionuclides—have been utilized in MRI imaging, fluorescence imaging, and radionuclide imaging to diagnose AR (Thiruppathi et al., 2017; Gao et al., 2021; Vazquez-Prada et al., 2021). Macrophages and T cells are the representative infiltrating cells involved in the rejection of a cardiac transplant. Their activities are closely associated with the progression of rejection, thereby making them ideal target cells for specific molecular imaging probes to diagnose the progression of heart transplant rejection by monitoring cell activities. Nanoparticles are extensively utilized in molecular imaging technology due to their small size, high surface area-to-volume ratio, and multifunctional properties. In addition, these nanoparticles can bind to specific sites and generate strong signals following the modification of ligands, thereby enabling the accurate detection of specific cellular structures. This demonstrates significant potential in the field of heart transplant diagnostics. In this section, we review and discuss different modalities of noninvasive imaging molecular probes—including ultrasound imaging, MRI, nuclide imaging, and fluorescence imaging—for diagnosing the occurrence of heart transplant rejection and evaluating the therapeutic effects of nanomaterials (Table 2).

**TABLE 2:** Targeted imaging molecular probes for diagnosing transplant rejection.

Target cells	Target marker	Probes	Imaging modality	References
T cell	CD25	NB_CD25_	US	Wu W et al. (2013)
T cell	CD3	NB_CD3_	US	Liu et al. (2018)
T cell	CD4	NB_CD4_	US	Xie et al. (2021)
T cell	GzB	MBGzb	US	Jin et al. (2023)
endothelial cells	ICAM-1	MB_ICAM_	US	Weller et al. (2003)
T cell	CD4	Cell-MB_CD4_	US	Xie et al. (2019)
Macrophages		USPIO	MRI	Wu YL et al. (2013)
Macrophages	Dextran-binding C-type lectins	dextran-coated USPIO	MRI	Berry et al. (2011)
T cell		IOPC-NH2	MRI	Liu et al. (2012)
T cell	CD3	ScAbCD3-PEG-g-PEI-SPION	MRI	Guo et al. (2012)
Macrophages	Siglec-1	^99m^Tc-SER-4	SPECT/CT	O'Neill et al. (2015)
Monocytes and macrophages	CCR2	^64^Cu-DOTA-ECL1i/^68^Ga-DOTA-ECL1i	SPECT/CT	Heo et al. (2021)
T cell	CD4	^99m^Tc-HYNIC-CD4mAb	SPECT/CT	Duneton et al. (2022)
Activated T cell	OX40 (CD134)	^89^Zr-OX40 mAb	SPECT/CT	Hirai et al. (2021)
T cell	CD8	NIR fluorohore (CyOH-CL)-self immolative linker petide (N-acetyl-lle-Glu-Pro-Asp)	FI	Gao et al. (2023)
T cell	CD8	Gold NP-petide (Biotin-SGSRSGIEFDKGGSGGG-NH_2_)	FI	Liu et al. (2022)

US, Ultrasound; NB, Nanobubble; MB, Microbubble; IOPC-NH2, Amination-modified superparamagnetic iron oxide nanoparticles; SPION, superparamagnetic iron oxide nanoparticles; USPIO, ultrasmall superparamag- netic iron oxide; PET, Positron Emission Tomography; SPECT, Single-Photon Emission Computed Tomography; CT, Computed tomography; MRI, Magnetic Resonance Imaging; FI, Fluorescence imaging; NIR, near-infrared.

### 3.1 Ultrasound molecular imaging

Ultrasonic molecular imaging is a noninvasive examination method that utilizes contrast agents to enhance scattering echoes with excellent resolution, sensitivity, and specificity. Microbubbles (MBs) are the most frequently used ultrasound contrast agents. However, they struggle to pass through the small gaps between the endothelial cells of newly formed blood vessels; to overcome this, nanoscale ultrasound contrast agents have been developed. These contrast agents comprise lipids, proteins, or the stability of the shell structure formed by polymers (Frinking et al., 2020). By labeling microbubbles and nanobubbles with targeted materials, specific disease markers can be effectively detected and quantified. A variety of biological targets for nanobubbles-targeted imaging have been proposed, such as cell adhesion molecules and other proteins related to cell recognition (Güvener et al., 2017).

**FIGURE 4:**
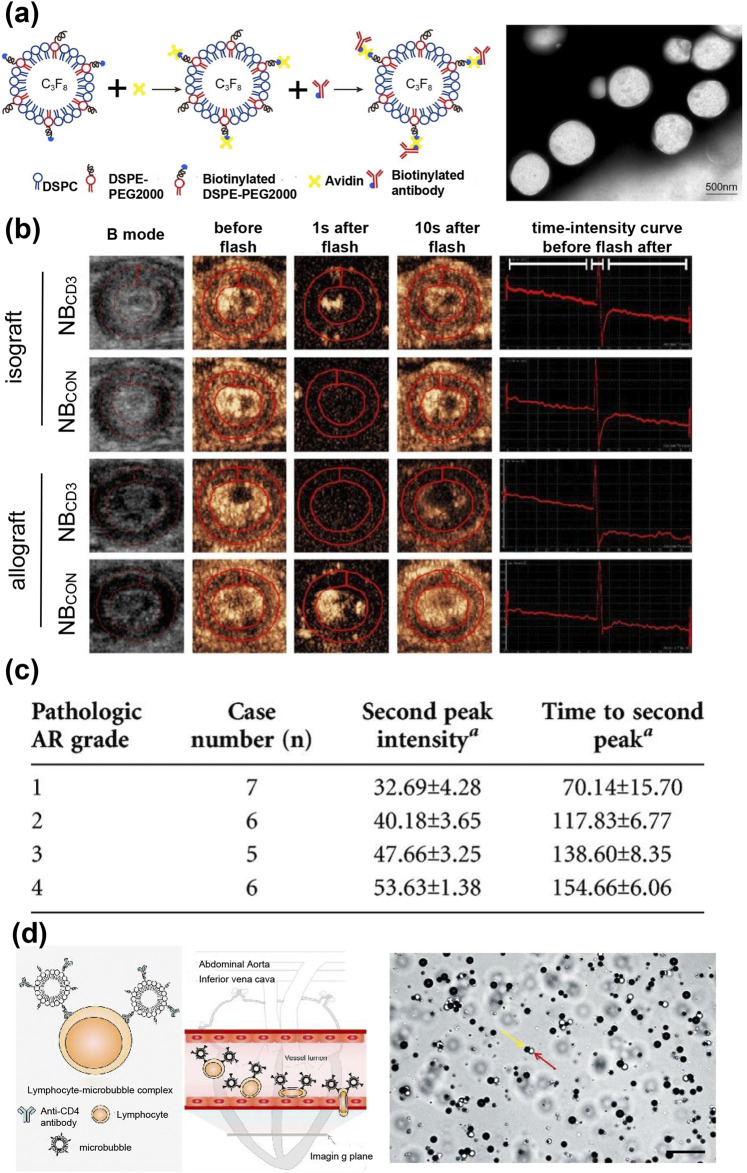
**(a)** Preparation of cd3-targeted nanobubbles (NB_CD3_) by biotin-avidin-biotin linking scheme; transmission electron microscope of NB_CD3_. **(b)** Representative ultrasound images of the allograft and isograft groups after injection of NB_CD3_ or NB_con_ (Liu et al., 2018). **(c)** Intensity and time of second peak of TIC in allografts with NB_CD25_ compared among different pathologic AR grades (Wu W. et al., 2013). **(d)** Diagram of the lymphocyte-microvesicle complexes (cell-MBs) and the interaction of cell-MBs with vascular endothelium in allogeneic grafts; representative bright field images of cell-MBs. The white dots (red arrows) are cells and the black dots (yellow arrows) are MBs (Xie et al., 2019).

T cell infiltration is the primary mechanism underlying AR. Studies have shown that nanobubbles targeting T cells can be used for ultrasound imaging and quantitative analysis of infiltrating T cells during rejection (Wu W. et al., 2013). For example, lipid nanobubbles containing the anti-CD3 antibody (NBCD3) have been prepared for targeted ultrasound molecular imaging of T cells (Figure 4a). The research found a positive correlation between the signal intensity of NBCD3 and the number of T-lymphocytes in the transplanted hearts of allo-transplanted rats (Figure 4b) (Liu et al., 2018). However, CD3-targeted imaging can identify any type of CD4 and CD8 T cells, including cytotoxic T cells and regulatory T cells. Xie et al. prepared CD4 antibody modified nanobubbles to target CD4 lymphocytes (Xie et al., 2021). The results indicated that the ultrasound signal intensity was correlated with the rejection grade and the number of infiltrating CD4 lymphocytes (Figure 4c). Granzyme B (GzB) is a protein that plays a related role in cytotoxic effects mediated by toxic lymphocytes, and its expression is significantly elevated during AR (Rowshani et al., 2004). Jin et al. conjugated biotinylated anti-GZB antibodies to streptavidin MBs to create targeted ultrasonic microbubbles for the quantitative assessment of granzyme B expression in cardiac grafts (Jin et al., 2023). Quantitative analysis revealed that the peak intensity difference in the MBGZB group was significantly greater compared to the allogeneic MBcongroup and the syngeneic MBcongroup. Furthermore, histological analysis indicated that the expression of granzyme B and CD8 T cell infiltration in the allogeneic group was higher than that in the allogeneic group, which was consistent with the results of the ultrasound. Similarly, Wu et al. prepared CD25 antibody-modified nanovesicles (NBCD25), where CD25 is a known marker for activated T cells (Wu W. et al., 2013). The NBCD25 ultrasonic signal was quantified into a time-intensity curve, and the intensity and time of delay enhancement and the second peak value could be used as quantitative indicators for the diagnosis and severity of AR. In addition, studies have revealed that ultrasound targeted microbubble destruction (UTMD) technology can promote the transfer of AR-related miRNAs from transplanted heart tissue to blood, thus achieving noninvasive early detection of AR (He et al., 2023).

Intercellular adhesion molecule-1 (ICAM-1), an inflammatory marker found in endothelial cells, is overexpressed in acute organ transplant rejection and facilitates leukocyte adhesion and infiltration (Wang et al., 1998). Weller et al. demonstrated the effectiveness of ICAM-1 targeted microbubbles in ultrasonic imaging of acute heart allograft rejection (Weller et al., 2003). Kondo et al. reported that leucocyte-targeted myocardial contrast-enhanced ultrasound could assess the extent of acute allograft rejection in rats (Kondo et al., 2004). But the increase in graft endothelial ICAM-1 and leukocyte infiltration is not a specific indicator of AR, and both processes also occur in ischemia/reperfusion injury (Lange et al., 2008). Lymphocytes are transported across the vascular endothelial barrier through lymphocyte-endothelial recognition and then infiltrate the interstitial tissue. If lymphocyte attraction and adherence of lymphocytes to cardiac endothelial cells can be detected, early diagnosis of acute cardiac cell rejection may be possible. Xie et al. developed T cell-MB complexes as ultrasound molecular imaging probes by co-incubating T cells with MBs conjugated to anti-CD4 antibodies (Figure 4d) (Xie et al., 2019). In an acute cell rejection environment, cell-MBs is drawn to endothelial cells at the site of cardiac rejection and engages in an adhesion cascade. Their results revealed that the ultrasonic molecular imaging signal of cell-MBs is enhanced with an increase in the rejection grade of a heart transplant. This ultrasonic molecular imaging of cell MB may offer a novel noninvasive technique for the early diagnosis of acute cardiac cell rejection. In addition, the infiltration of macrophages during graft rejection is closely related to the prognosis of the graft. Mannose modified MBs targeting CD206 (mannose receptor) positive macrophages can be used for the early diagnosis of chronic rejection (Xu et al., 2025).

### 3.2 Magnetic resonance imaging

**FIGURE 5:**
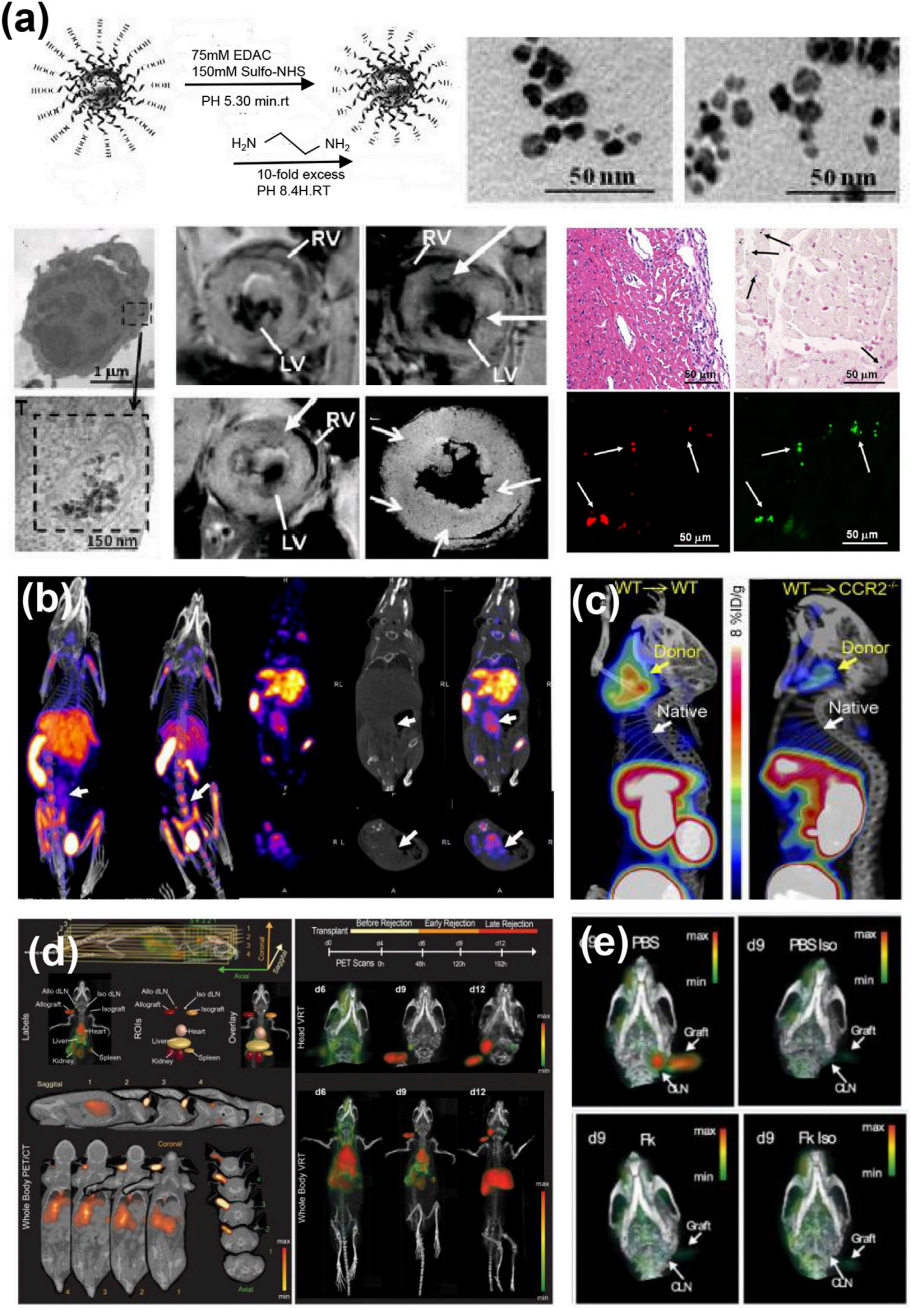
**(a)** Synthetic scheme of amine-modified superparamagnetic iron oxide nanoparticles (IOPC–NH2) and TEM images; TEM images of T-cells labeled with IOPC-NH2 particles; MRI imaging of IOPC-NH2 tracking of infiltrating T cells in transplanted hearts; immunohistochemistry and double immunofluorescence staining confirmed that the cells containing IOPC-NH2 particles are indeed T cells (Liu et al., 2012). **(b)** ^99m^Tc-SER-4 nano SPECT/CT imaging of Sn^+^ macrophages in allograft and isograft cardiac transplant model. Arrows indicate the site of the abdominal cardiac transplant (O'Neill et al., 2015). **(c)** ^64^Cu-DOTA-ECL1i PET/CT images revealing significant signal in transplanted heart (yellow arrow) and weak accumulation in native heart (white arrow) (Heo et al., 2021). **(d)** Three-dimensional PET/CT images and regions of interest (ROIs) of ^89^Zr-OX40 mAb in a heart allograft model. **(e)** Representative ^89^Zr-OX40 mAb PET/CT images of mouse heart grafts treated with PBS or immunosuppressant (Fk) on day 9 post-transplantation (Hirai et al., 2021).

Most *in vivo* MRI studies rely on superparamagnetic iron oxide (SPIO) or ultra-small superparamagnetic iron oxide (USPIO) nanoparticles and show excellent spatial resolution (Rezaei et al., 2024). Macrophages are the primary type of infiltrating cells in allografts during rejection, and graft infiltrating macrophages are closely associated with both short-term and long-term outcomes of organ transplantation (Kopecky et al., 2020; Chen et al., 2024). Previous studies have achieved *in vivo* tracking of macrophages by utilizing their phagocytic activity towards nanoparticles or targeting the cell surface receptors of macrophages (Kanno et al., 2001; Wu Y. L. et al., 2013; Heo et al., 2021). Kanno et al. used dextran-coated ultra-small USPIO particles to track macrophage infiltration associated with rat cardiac allograft rejection, and these nanoparticles were taken up by macrophages infiltrated within the cardiac grafts, with a significant reduction in MR signal (Kanno et al., 2001). However, this uptake is not unique to macrophages, and phagocytes such as dendritic cells can also absorb iron oxide particles. For T cells, Liu et al. modified SPION (IOPC-NH2) by amination and coated them with polyethylene glycol (PEG), which increased the labeling efficiency of T cells *in vivo* without affecting their proliferation and activation functions, and detected T cell infiltration at the allograft heart by *in vivo* MRI (Figure 5a) (Liu et al., 2012). In addition, further modifications with T cell-specific antibodies (such as CD3 monoclonal antibodies) can endow SPION with more precise targeting ability (Guo et al., 2012). With the ongoing advancement of nanotechnology, iron oxide nanoparticles are now utilized not only as contrast agents for diagnostic purposes but also as tracers in nanotherapeutic systems to monitor treatment effects and assess prognosis, which will be discussed in detail in the following article.

### 3.3 Nuclide molecular imaging

Unlike CT and MRI, which primarily display anatomical structures, radionuclide imaging reveals physiological functions, biochemical metabolism, and molecular pathway activities. It is dynamic and highly sensitive, capable of detecting extremely minute changes in biomolecules. The development of novel nuclear molecular imaging probes that specifically target immune cells may enhance the sensitivity and specificity of nuclear medicine imaging in detecting transplant rejection. Imaging pro-inflammatory macrophages infiltrating the transplanted organ is an effective method for monitoring the recipient’s immune response to the donor graft. Sialoadhesin (Sn, Siglec 1, or CD169) is a marker of inflammatory macrophages. Sn macrophages can activate various immune effector cells—including CD8 T cells, B cells, and iNKT cells—to enhance immune responses (Asano et al., 2011; Grabowska et al., 2021). O’Neill et al. used technetium-99 m radiolabeled anti-sialic adhesin monoclonal antibody SER-4 to develop a nuclide molecular probe (^99m^Tc-SER-4) for detecting inflammatory macrophages in rejection inflammation (Figure 5b) (O'Neill et al., 2015). The results indicated that ^99m^Tc-SER-4 signal was the strongest detected in the allogeneic heart transplantation model. Chemokine receptor-2-positive (CCR2^+^) monocytes and macrophages are recruited to the site of myocardial injury by chemokines and are important mediators of adverse inflammatory remodeling following heart transplantation. Based on this characteristic, Auvynet developed a short peptide extracellular ring one inhibitor (ECL1i) that exhibits allosteric and antagonistic effects on CCR2 (Auvynet et al., 2016). CCR2^-^ specific inflammatory load has been quantified in various animal models using this peptide-modified PET targeting tracer (Liu et al., 2017a; Li et al., 2018; Heo et al., 2019). Heo et al. utilized the ECL1i peptides to target and deliver ^64^Cu- and ^68^Ga-radiolabeled PET tracers (^64^Cu-Dota-ECL1i and ^68^Ga-Dota-ECL1i) to CCR2^+^ mononuclear cells/macrophages. The tracer uptake in donor hearts was four times that in autologous hearts in mouse heart transplantation models (Figure 5c) (Heo et al., 2021). In addition, the radioactive decay half-life of ^64^Cu is 10 times longer than that of ^68^Ga, and the ^64^Cu-DOTA-ECL1i tracer is currently approved by the U.S. Food and Drug Administration (FDA).

Regarding T cells, Li et al. developed a radionuclide molecular probe (^99m^Tc-HYNIC-CD4mAb) using CD47 monoclonal antibodies to monitor CD4^+^ T cells in rat allogeneic transplant hearts. The results indicated that the radioactive signals of callogeneic transplanted hearts significantly increased, consistent with the immunohistochemical findings. Treatment with cyclosporine leads to a reduction in the radioactive signal of allogeneic grafts, which correlates with a decrease in CD4^+^ T cell infiltration. However, while this nuclide probe can be used to monitor most T cells, it cannot discriminate the activation status of these cells. The costimulatory molecule OX40 (CD134) is a marker for activated T cells (Alam et al., 2020). Hirai et al. developed a nuclide probe (^89^Zr-OX40 mAb) using the radioactive element ^89^Zr-labeled OX40 antibody to monitor activated CD4^+^ T cells in the hearts of allografted mice. The results indicated that the ^89^Zr-OX40 mAb probe successfully detected activated CD4^+^ T cells that infiltrated allogeneic transplanted hearts during the early stages of the rejection response (Figures 5d,e). In addition, the immune PET signal in this study can also be utilized to assess the therapeutic effects of immunosuppressive drugs, thereby demonstrating significant potential for noninvasive monitoring of organ transplant rejection (Hirai et al., 2021).

### 3.4 Fluorescence imaging

Compared to traditional imaging methods, fluorescence imaging offers advantages such as real-time imaging, cost-effectiveness, noninvasiveness, and high spatiotemporal resolution. To date, fluorescent probes based on nanomaterials have been used to target cells or tissues across various disease models. The luminescent properties of these fluorescent molecules are employed to observe and analyze the conditions of human tissues or organs, enabling disease diagnosis and monitoring (Mateos et al., 2020; Gao et al., 2024).

**FIGURE 6:**
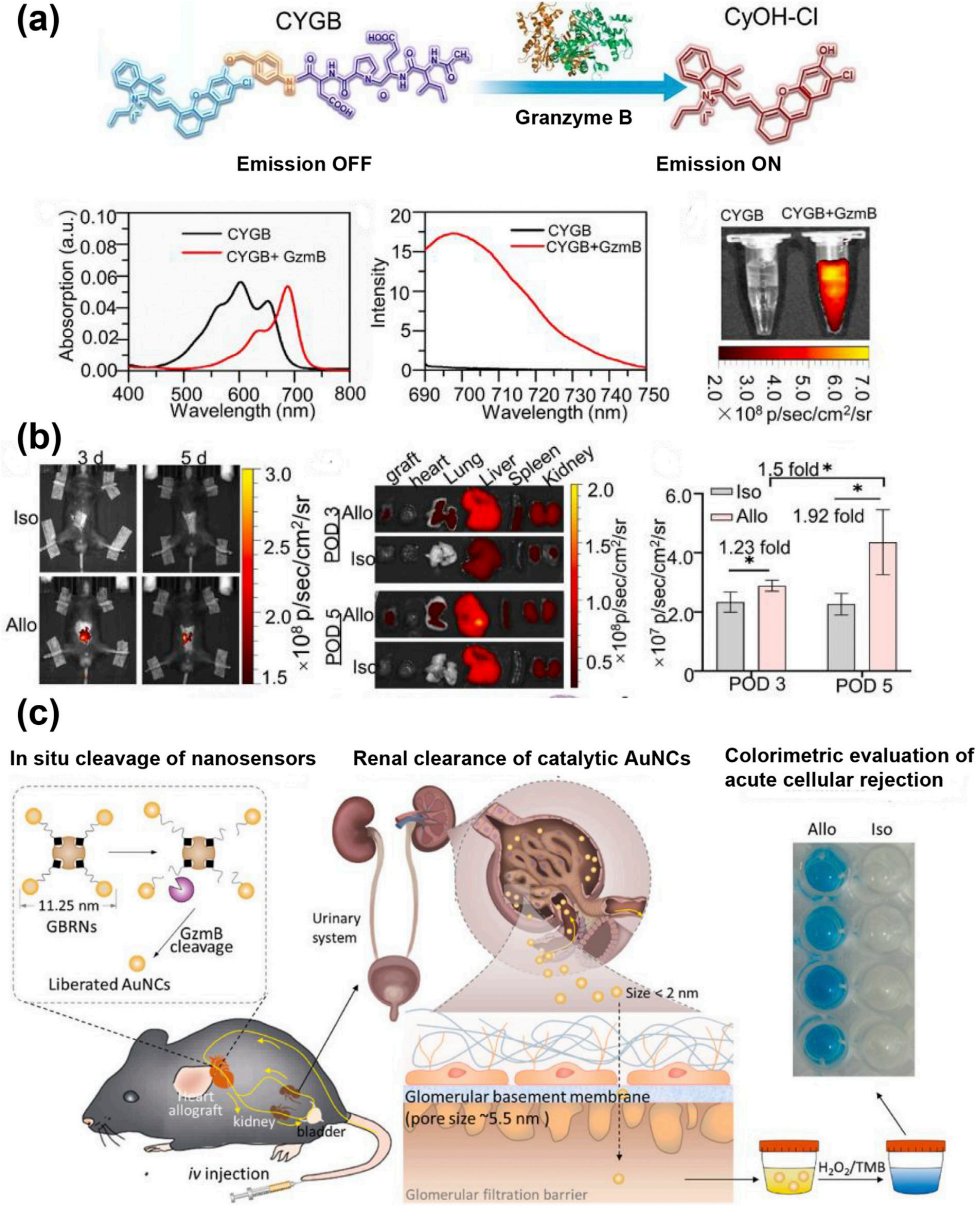
**(a)** Designing strategy of fluorescent probe, CYGB, for granzyme B; absorption and fluorescence emission spectra of CYGB (10 μM) in the absence or presence of Gzm B (0.225 U); corresponding fluorescence images obtained using the PerkinElmer IVIS imaging system (λex/em = 660/710 nm). **(b)** A whole-body NIR fluorescent imaging was performed for allografted and isografted mice; *ex vivo* fluorescence imaging of major organs from heart transplanted mice; quantitative fluorescence analysis of the grafted heart; data are presented as mean ± SD; n = 3, *p < 0.05 (Gao et al., 2023). **(c)** GzmB- Response nanosensors detect acute rejection (AR) in heart transplants by producing direct colorimetric urine readings (Liu et al., 2022).

During rejection, CD8^+^ T cells differentiate into cytotoxic T lymphocytes under inflammatory conditions. These cells then exert their cytotoxic effects through the secretion of perforin and granzyme B (Walch et al., 2013). Among these, the elevated expression of granzyme B has been demonstrated to be a valuable biomarker for monitoring rejection (Suthanthiran et al., 2013). Gao et al. prepared a granzyme b-reactive fluorescent probe, CYGB, for the early noninvasive diagnosis of transplant rejection (Figure 6a) (Gao et al., 2023). CYGB probes include a cage-type hemianthocyanin fluorophore and a granzyme b-specific cleavage substrate N-acetyl-Ile-Glu-Pro-Asp (IEPD) peptide. IEPD peptide sequences can be cleaved by granzyme B and are commonly used to design granzyme response probes. Fluorophores and peptides are linked by the self-cleavage of aminobenzyl alcohol. When granzyme B cleaves the peptide substrate at the site of transplantation rejection, a cascade of self-elimination spacers is triggered, which results in the release of a cage-like fluorophore and the emission of fluorescence. *In vivo* imaging studies have confirmed that the CYGB probe can enable the early diagnosis of skin and heart graft rejection in mice (Figure 6b). Furthermore, the study further validated the ability of the probe CYGB to assess immunosuppressive treatments in real time. However, optical imaging has the limitation of poor tissue penetration. To address this issue, researchers developed a direct colorimetric urine reading methods to study the activity of serine protease enzyme B particles. Liu et al. conjugated a granzyme B substrate (Biotin-SGSRSGIEFDKGGSGGC-NH2) with gold nanoparticles to synthesize granzyme B-responsive nanosensors (GBRNs) (Liu et al., 2022). The GBRNs that reached the allogeneic transplant heart were cleaved by granzyme B, and the gold nanoparticles were filtered into the urine through the glomeruli (Figure 6c). These gold nanoparticles have peroxidase-like activity, which results in the discoloration of the peroxidase substrate (3,3′,5,5′-tetramethylbenzidine). This granzyme B-responsive nanosensor can noninvasively detect transplant rejection through colorimetric urine readings within 1 hour. Targeted fluorescence imaging is a crucial biomedical imaging technology, but it is susceptible to interference from non-specific fluorescence and fluorescence quenching, and both imaging depth and tissue penetration are restricted.

Precise imaging and early diagnosis are vital for addressing cardiac transplant rejection, while the ultimate goal remains effective therapeutic intervention. Due to their unique size effects and design flexibility, nanomaterials serve as exceptional “diagnostic probes” and demonstrate significant potential as multifunctional “therapeutic carriers.” The construction of nanoscale drug delivery systems is expected to overcome the inherent targeting, solubility, and toxicity limitations of traditional drug administration methods, offering innovative solutions for treating various complex diseases. The following discussion elaborates on the design principles, functional modifications, drug loading methods of nanocarriers and their applications in treating cardiac transplant rejection, highlighting their profound implications for advancing modern medicine.

## 4 Nanotechnology for the treatment of heart transplant rejection

Immunosuppressive agents currently in clinical use target the adaptive immune system, such as calmodulin phosphatase inhibitors (cyclosporine and tacrolimus), cell cycle inhibitors, corticosteroids, and Mammalian rapamycin target (MTOR) inhibitors (Deng et al., 2020). While immunosuppressive therapy can significantly reduce the risk of rejection, it can also lead to a range of side effects and toxicities, such as an increased risk of infection, osteoporosis, and cardiovascular problems. In recent years, the use of nanoparticles as drug carriers to target regulatory cells has demonstrated significant effectiveness in the treatment of organ transplant rejection (Table 3). As drug carriers, nanoparticles offer significant advantages in improving the stability, biocompatibility, and targeting properties of drugs, while also reducing adverse reactions. The development of various specific targets further enhances the efficacy of nanomedicine. In addition, nanoparticles that respond to stimuli and enable controlled release have been developed based on changes in the microenvironment at the site of rejection lesions (Lu et al., 2024). These targeted delivery methods enhance therapeutic efficacy by precisely modulating the responses of target cells and organs, while simultaneously reducing the total dose of immunosuppressive drugs, thereby mitigating drug side effects and complications.

**TABLE 3:** Nanomedicine for the treatment of transplant rejection.

Target organ	Tageted molecules /cells	Drug	NP composition	ROA	Ref
Lymph node	CD3^+^ T cell	FK506	PEG-PLGA NP	iv	Shin et al. (2010)
Lymph node	CD3^+^ T cell	FK506	PLGA NP	sc	Deng et al. (2021)
Lymph node	CD8^+^ T cell	PP242	Docosahexaenoic acid	ip	Zhou et al. (2021)
Lymph node	CD3^+^ T cell	FK506	β-glucan microcapsules	po	Wu Y et al. (2020b)
Lymph node	Treg cell	FTY720	CaCO3/CaP/Heparin Hybrid carrier modified with CCL21	iv	Che et al. (2023)
Lymph node	PNAd	CD3 antibody	MECA79 antibody - PLGA NP	iv	Bahmani et al. (2018)
Lymph node	PNAd	CD40L antibody	MECA79 antibody - PEG-PLGA	iv	Zhao et al. (2022)
Lymph node	PNAd	FK506	MP-FK506-MECA79	iv	Azzi et al. (2016)
Graft heart	CD3^+^ T cell	FK506	LP	UTMD	Liu et al. (2019)
Graft heart	LRP1-CALR	RAPA	FNVs@RAPA	iv	Deng et al. (2020)
Graft heart	HIF-2α-CSF1R	roxadustat	Roxadustat-loaded micelle	iv	Fishbane et al. (2021)
Graft heart	T cell	MMF	PEG-PLGA NP	ecp	Hussain et al. (2021)
Graft heart	T cell	MTX	LDE-MTX	iv	DeBerge et al. (2024)
Graft heart	T cell	PACLI	LDE-PACLI	iv	Fiorelli et al. (2017)
Graft heart	CD3^+^ T cell	Antagomir-155	Lipid microbubble	UTMD	Yi et al. (2020)
Graft heart	CD3^+^ T cell	Antagomir-155	Lipid microbubble	GVs+ ultrasound	Gracias et al. (2013)
Graft heart	Th cell	Galectin-7-siRNA	Lipid microbubble	UTMD	Wang et al. (2020)
Graft heart	PD-L1/PD-1 CTLA-4/CD80	PD-L1/CTLA-4	PD-L1/CTLA-4 NVs	iv	Xu et al. (2020)
Graft heart	T cell	PD-L1	PD-L1 Exo	iv	Luo et al. (2023)
Graft heart	T cell	FK506 FGL1/PD-L1	sEVs@FK506	iv	Tsai et al. (2022)
Graft heart	BMDCs	RAPA TRAF6i	89Zr-mTORi-HDL TRAF6i-HDL	iv	Braza et al. (2018)
Graft heart	CD3^+^ T cell	DKGα gene	CD3 antibody-modified SPION	iv	Guo et al. (2012)
Graft heart	CD4	NO	NO-MB_C4d_	iv	Liao et al. (2020)

FK506, Tacrolimus; FTY720, fingolimod hydrochloride; RAPA, Rapamycin; MMF, mycophenolate mofetil; iv, Intravenous injections; ecp, Extracorporeal perfusion; UTMD, Ultrasound-targeted microbubble destruction; ip, Intraperitoneal injections; sc, Subcutaneous injections; po, Oral administration; Treg cell, regulatory T cells; Th cell, T helper cells; BMDCs, bone marrow-derived dendritic cells; MPA, Mycophenolic acid; EVs, extracellular vesicles; Exo, exosomes; NVs, nanovesicle; FNVs, fusion extracellular nanovesicles; LDE, Lipid nanoparticles; PNAd, peripheral lymphonode vascular addressin; LP, liposomes; PLGA, poly(lactic-co-glycolic acid); NP, Nanoparticle; MP, microparticles; PEG-bl-PPS, Poly(ethylene glycol)-bl-poly(propylene ulfide); PEG, polyethylene glycol; SPION, superparamagnetic iron oxide nanoparticles; GVs, gas vesicles. HDL, high-density lipoprotein. ROA, route of administration.

### 4.1 Nanoparticles target lymph nodes to treat heart transplant rejection

Lymph nodes (LNs) play a crucial role in immune activation and regulation, and delivery of targeted drugs to LNs has been shown to be an effective method of enhancing transplantation immunity. The most common approach involves using polylactic acid-glycolic acid (PLGA) nanoparticles loaded with immunosuppressive drugs. Compared with free FK506, FK506-loaded poly (ethylene glycol) methyl ether-block-poly (lactide-co-glycolide) (PEG-PLGA) and PLGA nanoparticles (PEG-PLGA-FK506 and PLGA-FK506) showed higher accumulation in rat mesenteric and axillary lymph nodes after intravenous injection and successfully attenuated acute rejection and prolonged survival of allogeneic grafts (Shin et al., 2010). PEG-modified PLGA nanoparticles enhance the stability and water solubility of drugs, reduce phagocytosis by the mononuclear phagocytic system, and prolong the circulation time of drugs in the body (Suk et al., 2016). In addition, the subcutaneous (SC) administration of PLGA nanoparticles loaded with FK506 (PLGA-FK506-NPs) can effectively deliver FK506 to the LNs (Deng et al., 2021).

**FIGURE 7:**
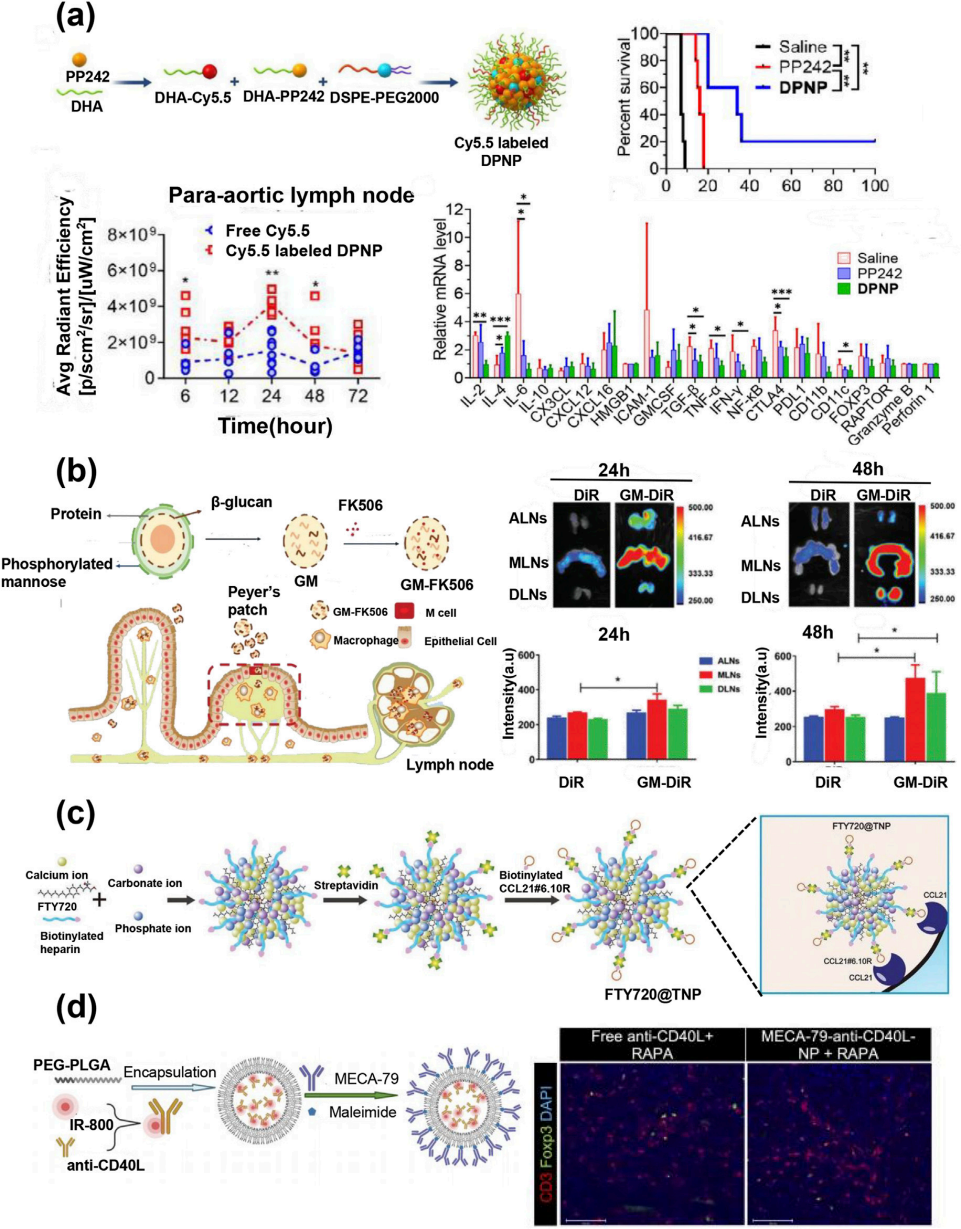
**(a)** Synthesis procedure for Cyanine5.5 (Cy5.5)-labeled DHA-PP242 nanoparticles (DPNP); quantification of Cy5.5 fluorescence intensity in para-aortic lymph nodes; survival time of heart transplant recipients in different treatment groups; relative mRNA expression of immune-related genes in grafts (Zhou et al., 2021). **(b)** Schematic representation of β-glucan microcapsules-FK506 (GM-FK506) preparation and lymph node targeting mechanisms; fluorescence images and quantitative analysis of lymph nodes *in vitro* after oral GM-DiR (Wu Y. et al., 2020). **(c)** Preparation of FTY720-loaded targeting nanoparticles (FTY720@TNP) with CCL21#6.10R (Che et al., 2023). **(d)** Schema of anti-CD40L–IR-800 synthesis and conjugation with MECA-79 mAb; representative fluorescence micrographs of CD3^+^ T cells and Foxp3^+^ Tregs in allograft hearts (Zhao et al., 2022).

In addition to PLGA nanoparticles, other nanomaterials have also been used to deliver immunosuppressants to lymph nodes. For example, Zhou et al. conjugated the rapamycin complex kinase inhibitor PP242 with polyunsaturated fatty acids to form self-assembled nanoparticles (Zhou et al., 2021). Following systemic administration, the DPNPs demonstrated the ability to accumulate in the lymph nodes and spleen, thus effectively prolonging the survival time of transplanted hearts. The potential mechanism of this process involves promoting the accumulation of DPNP in macrophages, impairing the function of M1 macrophages, inhibiting their production of IL-6, and subsequently reducing the number of types I CD4 T cells and CD8 T cells (Figure 7a). Research reported that β-glucan particles extracted from yeast are recognized and phagocytosed by macrophages in the Peyer’s patches of the intestinal wall, followed by transport to mesenteric lymph nodes (Aouadi et al., 2009). In addition, Wu et al. loaded FK506 into β-glucan microcapsules (GMs). Following oral administration, the GM microcapsule was bound to the Dectin-1 receptor on the surface of macrophages and continuously released for 48 h, thus effectively delivering FK506 to the lymph nodes (Figure 7b). This delivery method significantly improved the survival rate of allografts, notably reduced the grade of AR, and no renal toxicity was observed after continuous administration (Wu Y. et al., 2020). Mu et al. developed a novel FK506 nanomedicine (FK506 cochleates) using microfluidic methods to reduce variability among individuals and improve drug safety. After oral administration, FK506 cochleates accumulated in the spleen and lymph nodes, significantly prolongs survival time (Mu et al., 2024). Oral nanomedicines demonstrate significant potential in enhancing safety and improving patient compliance. However, although a few oral nano-medicines have successfully entered the market, they still face significant challenges in production processes, costs, safety, and complex biological barriers. To achieve Widespread application, sustained efforts from both the scientific community and the industrial sector are required.

With the advancement of nanotechnology, researchers have altered the surfaces of nanoparticles using chemical or biological methods to specifically bind to receptors or other cytokines that are highly expressed in target organs, further improving the efficacy of nanoparticle-targeted drug delivery. For example, chemokine CCL21 specific aptamers were modified on the surface of heterogeneous nanoparticle delivery carriers composed of CaCO3/CaP/heparin to deliver immunosuppressant FTY720 hydrochloride, which achieved LN specific immune regulation and weakened the immunosuppression of distant peripheral immune organs (Figure 7c). This led to effectively prolonging the survival time of the mouse heart transplantation model (Che et al., 2023). Tannic acid (TA) molecules exhibit strong affinity for the elastic fibers of LNs conduits. TA-modified FK506-loaded PEG-PLGA nanoparticles can traverse the intercellular gaps in the lymphatic endothelial cell layer, enter the paracortex through the capsule-associated conduits of lymph nodes and accumulate within the lymph nodes. The activation and proliferation of T cells were inhibited, and the rejection grade of the transplanted heart was significantly reduced (Qiu et al., 2025).

High endothelial venules are a specialized component of the LN vasculature and uniquely express peripheral lymph node addressing (PNAd) molecules, which are recognized by the MECA-79 monoclonal antibody (mAb). Bahmani et al. covalently bound MECA79 mAb to the surface of PLGA-PEG nanoparticles as a carrier of CD3 antibodies to achieve the targeted delivery of LN (Bahmani et al., 2018). The results revealed a significant increase in the number of TreGs *in vivo* and in DLNs in mouse heart transplant models, which led to an extended survival time of the allografts. Similarly, the CD40/CD40L pathway plays a crucial role in regulating adaptive immune responses (Webb et al., 2016). The selective delivery of anti-CD40L-loaded nanoparticles (anti-CD40L-NPs) to LNs by coating MECA-79 (MECA-79-anti-CD40L-NPS) significantly delayed the occurrence of cardiac allograft rejection and increased the number of Tregs (Figure 7d) (Zhao et al., 2022). In addition to loading antibodies, polylactic acid (PLA) particles modified with MECA-79 monoclonal antibodies were utilized to target the immunosuppressive drug FK506 (MP-FK506-MECA79) to lymph nodes, thereby effectively reducing the risk of heart transplant rejection (Azzi et al., 2016).

### 4.2 Nanoparticles target transplanted organs to treat heart transplant rejection

**FIGURE 8:**
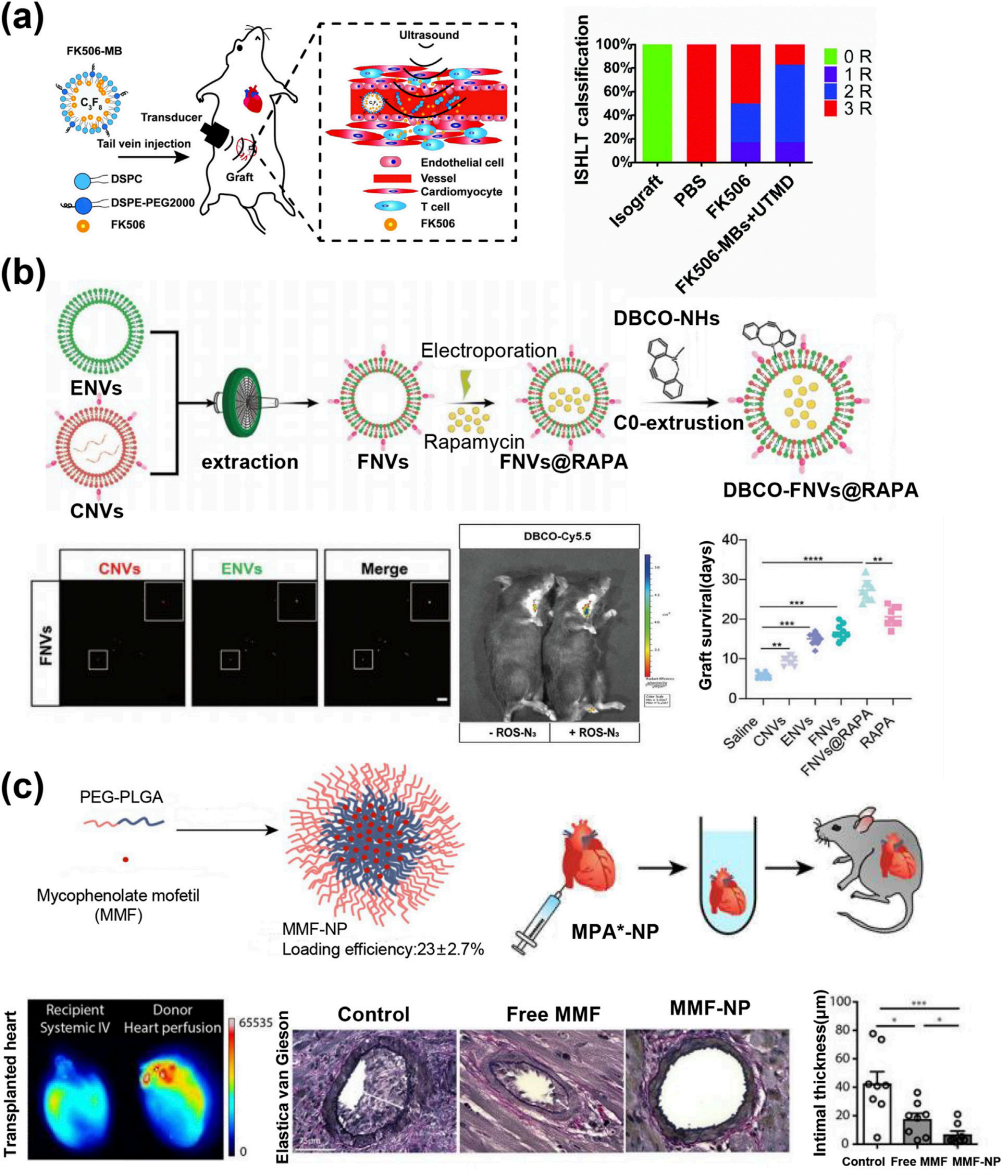
**(a)** Schematic diagram of the *in vivo* treatment process of FK506-MBs + UTMD; AR grades of each treatment group (Liu et al., 2019). **(b)** Schematic representation of the preparation of DBCO-FNVs@RAPA; confocal images of FNVs; *in vivo* imaging of DBC0-Cy5.5 distribution in ischemia-reperfusion transplanted mice; survival days of transplanted heart after different treatments; n = 9, **p < 0.01, ***p < 0.001, ****p < 0.0001 (Deng et al., 2020). **(c)** Schematic of MMF-NP fabrication; MMF-NP was perfused into the donor heart, and the perfused heart was transplanted into the recipient mouse; MPA*-NP signal was significantly higher in the donor cardiac perfusion group than in the recipient systemic intravenous group; Elastica van Gieson staining of the allograft heart and quantitative intimal thickness (Hussain et al., 2021).

Nanoparticles designed to target transplanted organs significantly enhance the efficacy of medications while minimizing toxic side effects. During a graft rejection reaction, the local vascular permeability of transplanted organs increases and this leads to the passive accumulation of nanoparticles. This process can enhance the concentration of drugs within the transplanted organ (Suzuki et al., 2010; Liu et al., 2019). Microvesicles are not only ideal contrast agents for ultrasound imaging, but also excellent drug carriers for treatment. Ultrasound-targeted microbubble destruction (UTMD) enhances the efficiency of drugs carried by microbubbles crossing biological barriers and is used for drug delivery in the treatment of various diseases (Yang et al., 2025; Zhang et al., 2025). In a heart transplantation model, the use of an ultrasonic beam focused on the heart induces a cavitation effect that triggers the oscillation and collapse of microbubbles. This process enhances the endothelial barrier and increases cell membrane permeability, thereby improving drug exosmosis. Liu et al. demonstrated that using UTMD for the selective delivery of FK506 microbubbles promoted the distribution of the drug in transplanted heart tissues, thereby reducing heart transplant rejection (Figure 8a) (Liu et al., 2019). In addition, ultrasound-targeted destruction of cationic microbubbles carrying therapeutic genes (siGal-7/Antagomir-155) enables selective transfection of cardiomyocytes to silence the expression of related proteins, alleviated the rejection reaction of heart transplantation (Wang et al., 2020; Yi et al., 2020) Similar to UTMD technology, sonodynamic therapy also possesses ultrasonic targeting capabilities. Li et al. employed MCP-1 peptides to target and induce cardiac-resident CCR2^+^ macrophages to phagocytize nanoparticles carrying sonosensitizers. Then, in combination with low-intensity focused ultrasound, the sonosensitizers were activated to generate reactive oxygen species (ROS) to induce apoptosis, thereby inhibiting subsequent rejection reactions (Li et al., 2026).

Macrophage membrane-biomimetic nanoparticles retain the inflammatory targeting and immune evasion capabilities of macrophages. For instance, Bao et al. prepared macrophage membrane-modified nanoscale coordination polymers (dNCPs@MM) through coordination-driven self-assembly of the immunosuppressant dexamethasone sodium phosphate (DEXp) with Fe^3+^. These nanopolymers exhibited responsive release characteristics and targeted homing to transplanted hearts, significantly enhancing biological efficacy, promoting immune tolerance, and prolonging the survival time of transplanted mice (Bao et al., 2025). Additionally, leveraging the characteristic infiltration of macrophages into transplanted hearts, targeting circulating inflammatory monocytes/macrophages and “hitchhike” to the transplanted heart is another promising strategy to improve the targeting of nanomedicines. At present, most nanomedicines are administered by intravenous injection, frequent administration of drugs can impose a huge psychological burden on patients. To overcome the shortcomings of systemic administration, researchers have prepared adjustable and biodegradable long-acting microfibers. When implanted subcutaneously in the abdomens of transplanted rats, these microfibers enabled sustained release of the highly hydrophobic FK506 over an extended period, successfully mitigating acute cardiac transplant rejection (Deng et al., 2023). In addition, myocardial injection of drug-loaded gel is also one of the effective methods for local treatment of transplanted hearts (Majumder et al., 2020). In recent years, nano-smart patches directly acting on the heart have achieved remarkable results in the research related to myocardial infarction repair (Qiu et al., 2024; Wang et al., 2025). However, most of them are still in the research stage at present, and their long-term safety and clinical efficacy need to be further verified.

Monocytes recruited during the immune response of transplanted organs differentiate into pro-inflammatory (M1) and anti-inflammatory (M2) macrophages under the action of local cytokines (Pascual-Gil and Epelman, 2018; Arnold et al., 2019; Jiao et al., 2023). Promoting the transformation of cardiac macrophages to M2 type is considered as a potential method to reduce rejection damage. To improve the targeted delivery of Rapamycin (RAPA) to M1 macrophages in the transplanted heart, researchers used multifunctional plant-animal hybrid nanovesicles as natural nanocarriers. Through genetic engineering, calreticulin (CALR, an “eat me” signal) was expressed on the surface of RAPA-carrying EVs to promote their uptake by M1 macrophages (Zhang Y. R. et al., 2020). Simultaneously, leveraging the characteristic of transplanted hearts releasing high levels of reactive ROS during IRI, the researchers designed ROS-responsive Ac4ManAz derivatives. These derivatives enable targeted drug delivery at the heart transplant site via strain-promoted click chemistry, effectively alleviating IRI, promoting polarization toward M2 anti-inflammatory macrophages, prolonging graft survival, and confirming treatment safety (Figure 8b) (Lu et al., 2024). Roxadustat-encapsulated PEG-PPS micelles target bone marrow cells, promoting the upregulation of HIF-2α and its transcriptional target colony-stimulating factor 1 receptor (CSF1R) in splenic monocytes. HIF-2α mobilizes specific tolerogenic monocytes in the spleen, while CSF1R mediates the differentiation of monocytes into tolerogenic macrophages. The results revealed that the survival rate of cardiac allografts increased in the experiment (DeBerge et al., 2024). These data support further exploration of HIF-2α activation in myeloid cells as a therapeutic strategy for promoting transplant tolerance.

Many natural materials found in the body are also used as nanocarriers to transport drugs to transplanted hearts, such as albumin, lipoproteins, and dopamine. These materials exhibit superior biocompatibility and can play a unique role through specific physiological and biochemical processes. For example, studies have revealed that the gene expression of lipoprotein receptors is significantly higher in grafts compared to native hearts. Stolf et al. used low-density lipoprotein analogues (LDEs) as nanocarriers to deliver the chemotherapeutic drugs methotrexate (MTX) and paclitaxel (PACLI) (Barbieri et al., 2017; Fiorelli et al., 2017). Pharmacokinetic studies have shown that LDEs are four times more concentrated in transplanted hearts than in orthotopic hearts and greatly increase the cellular uptake of drugs. Rabbit models of heart transplantation treated with LDE-MTX reduced the secretion of TNFα, monocyte chemoattractant protein 1, and IL-18; increased the secretion of the anti-inflammatory factor IL-10; and significantly improved vasculopathy and inflammation in cardiac allografts (Fiorelli et al., 2017). In addition, treatment with LDE-PACLI resulted in reduced macrophage infiltration and significantly lower coronary artery stenosis in the rabbit model than in the PACLI group (Barbieri et al., 2017). The surface of these natural nanoparticles can also be modified by materials such as antibodies or targeting ligands to further enhance their functions, which has great application prospects, but it is still rarely used in the field of heart transplantation.

Further, the time interval between the collection of organs from the donor and their implantation into the recipient presents a unique clinical setting for organ transplantation. This period offers an ideal opportunity for the localized, continuous, and controlled delivery of nanomedicine, thereby acting more effectively on endothelial cells and the transplanted organ (Hussain et al., 2021). Relevant studies have demonstrated that controlling early immune activation in transplanted organs and reducing their immunogenicity can minimize the need for immunosuppression. Uehara et al. developed a PEG-PLGA nanoparticle (MMF-NPS) containing mycophenolate mofetil (MMF) to infuse mouse hearts prior to transplantation (Figure 8c) (Uehara et al., 2019a). The results revealed that the NP signal was stronger in the transplanted heart and lower in other major organs with external perfusion compared with intravenous injection. In addition, the expression of pro-inflammatory cytokines and chemokines in allogeneic transplant hearts perfused with MMF-NP was significantly reduced, thus effectively preventing vascular pathology associated with heart transplants. Targeting peptide-modified rapamycin micelles (TRaM) added to organ preservation solution can inhibit endothelial and epithelial activation in allografts, which significantly reduces organ damage and induces a tolerogenic graft microenvironment (Zhu et al., 2018). Furthermore, local delivery of anti-IL-6 nanoparticles not only protects transplanted hearts from IRI but also significantly mitigates chronic immune rejection (Solhjou et al., 2017). Such a pre-transplant organ nanodrug delivery system has also been used to pretreat endothelial cells or grafted skin from human transplanted vessels, thereby significantly improving transplant outcomes (Cui et al., 2017; Uehara et al., 2019b). This method of delivering therapeutic drugs to donor organs via nanoparticles prior to transplantation not only improves the effect of the drug but also reduces the impact of the drug on other organs, which is promising for clinical conversion. It also facilitates the preservation and resuscitation of donor hearts, expands the donor organ pool, and holds promise for clinical translation.

### 4.3 Nanoparticles-targeted delivery of therapeutic genes to treat transplant rejection

Over the past decade, small non-coding RNAs (microRNAs) that regulate biological activity by regulating mRNA transcription have received a lot of attention. Numerous studies have demonstrated that transplant rejection is regulated by the gene in question, indicating the potential for gene therapy in the treatment of organ transplant rejection (Duong Van Huyen et al., 2014; Zhang et al., 2019; Zhang et al., 2023). Gene therapy uses nucleic acid drugs (e.g., small interfering RNAs (siRNAs), single guide RNAs (sgRNAs), and short hairpin RNAs (shRNAs) to bind to the mRNA of the target gene, causing the mRNA to degrade and, thus, inhibiting the expression of specific genes and their role in the treatment of disease (de Ramon et al., 2015; Brüggenwirth and Martins, 2020). However, efficiently delivering therapeutic genes to protein synthesis sites presents numerous challenges, including the fragile nature of genes, cellular barriers, immune stimulation, and off-target effects (Wang C. et al., 2023). To address these challenges, researchers have developed cationic nanoparticles and nanovesicles as delivery carriers. These materials form nanoscale complexes with negatively charged nucleic acids, which enhance the stability and tissue specificity of therapeutic genes *in vivo* and improve their therapeutic efficacy.

**FIGURE 9:**
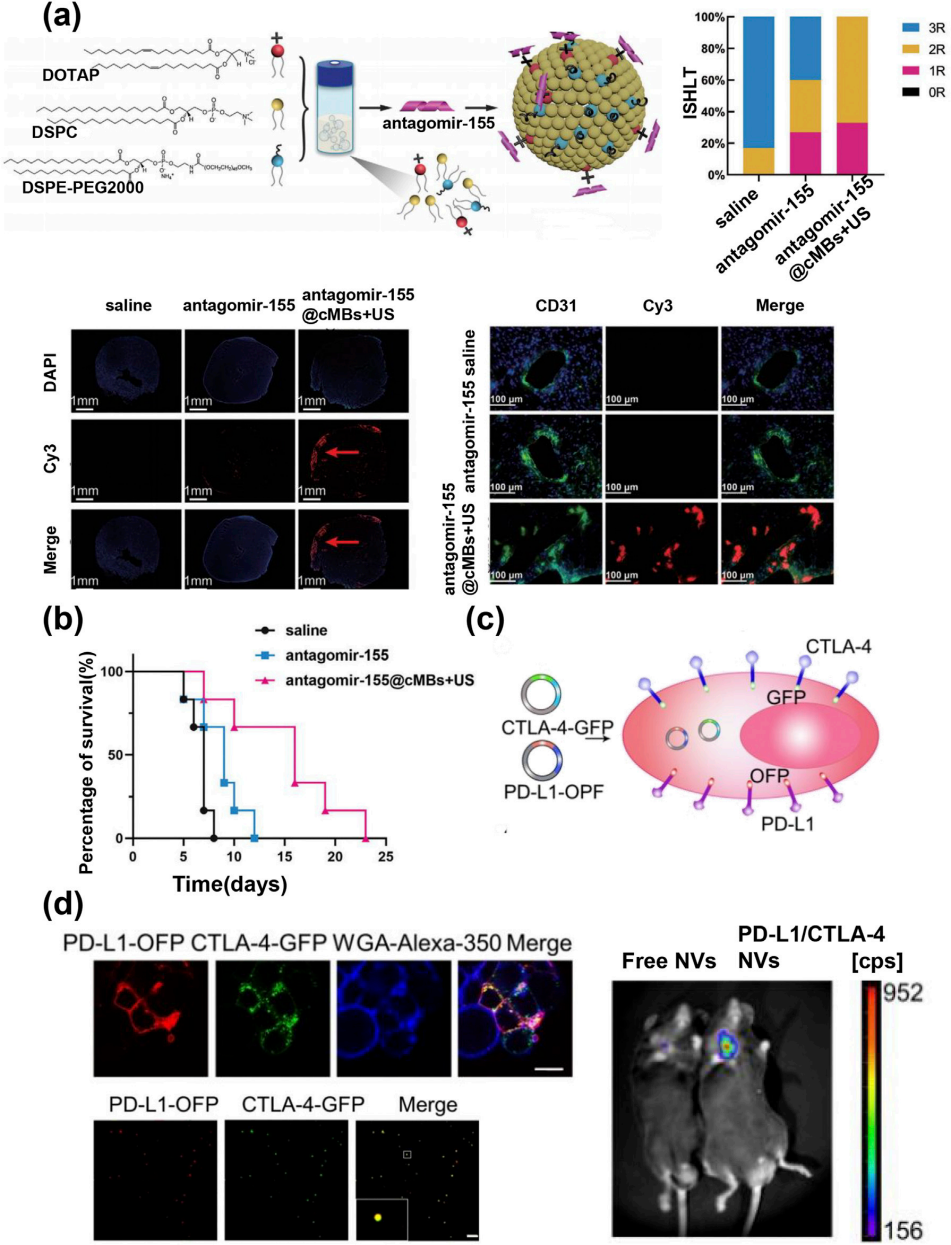
**(a)** Synthesis of antagomir-155 loaded cationic microbubbles; grade of rejection in different groups; antagomir-155 was delivered into allograft heart by UTMD; the fluorescence-labeled antagomir-155 was mainly located directly around the capillaries. **(b)** The allograft heart survival time (Yi et al., 2020). **(c)** The establishment of cells expressing PDL1/CTLA-4 dual targets with fluorescent labeling retained in the cell. **(d)** Confocal images verified the cell membrane localization and co-localization of PD-L1-OFP and CTLA-4-GFP in the same nanovesicles; PD-L1/CTLA-4 NVs accumulate intensively at the heart transplant site in the neck compared to free NVs (Xu et al., 2020).

MiR-155 is significantly increased in the serum of patients experiencing cardiac allograft rejection. Inhibiting the expression of miR-155 in mice reduces ACR (Duong Van Huyen et al., 2014). In addition, MiR-155 plays a crucial role in the activation of immune cells, their differentiation, antibody production, and the generation of cytokines, suggesting that miR-155 may be a potential therapeutic target for AR (Xu et al., 2022). Antagomir is a special chemically modified miRNA antagonist. Cationic MBs (CMBs) have been used to carry antagomir-155 through electrostatic adsorption; this is combined with the cavitation effect of UTMD to open cellular and vascular biological barriers (Figure 9a), locally release therapeutic genes, downregulate miRNA-155 expression in mouse allogeneic transplant hearts, and alleviate the AR of heart transplants (Figure 9b). (Yi et al., 2020) Similarly, galectin-7 promotes the rejection of transplanted hearts by facilitating the differentiation of helper T-cells to Th1 differentiation (Luo et al., 2018). Wang et al. prepared lipid microbubbles charge-coupled to galectin-7-siRNA and used UTMD to inhibit galectin-7 expression by siRNA-mediated knockdown in a rat abdominal ectopic heart transplantation AR model, preventing early acute cell rejection after a heart allograft without the need for systemic immunosuppression (Wang et al., 2020).

UTMD technology has been widely used in the treatment of various diseases. However, it is limited by issues related to tissue penetration and potential tissue damage. To reduce the ultrasound intensity required for microbubble bursting while minimizing potential damage, Wang et al. developed novel gas vesicles (GVs) (Wang R. et al., 2023). GVs are gas-filled protein nanostructures that can be triggered to cavitate upon exposure to low-intensity pulsed ultrasound (LIPUS), generating mechanical effects that assist RNA in heart transplantation. They used liposomal nanoparticles to encapsulate antagomir-155 and employed GVs along with low frequency ultrasonic waves to create a cavitation effect that enabled the nanoparticles to penetrate the blood vessel wall and reach the allograft heart. This cavity-assisted gene therapy strategy after heart transplantation upregulates the expression of cytokine signaling suppressor protein 1 (SOCS1) by inhibiting miR-155 as well as reducing the infiltration of inflammatory cells and the release of inflammatory factors. Therefore, the rejection is reduced and the survival time of the transplanted heart is significantly prolonged. To date, an increasing number of genes related to heart transplant rejection have been discovered, and these genes are promising to be used as the target of nano gene therapy to promote the treatment of heart transplant rejection (Zhang et al., 2023).

### 4.4 Extracellular vesicles for the treatment of heart transplant rejection

Extracellular vesicles (EVs) are nanoscale bilayer structures composed of bioactive molecules, they are typically released from cells under both normal and pathological conditions. There are three main categories of these vesicles: microvesicles (150 nm–1,000 nm), exosomes (40 nm–150 nm), and apoptotic bodies (>1,000 nm). They carry various bioactive molecules from the mother cell—including proteins, lipids, DNA, and RNA—which reflect the pathophysiological conditions of the body (Maas et al., 2017; Celik et al., 2023). Therefore, multiple studies have already demonstrated that EVs can serve as biomarkers for the early detection of cardiac transplant rejection, enabling noninvasive monitoring and providing a basis for personalized treatment (Mirzakhani et al., 2019; Castellani et al., 2020; Korutla et al., 2024; Burrello et al., 2025). Furthermore, these nanoscale vesicles play a central role in pathophysiological processes such as disease development, immune response, and tissue repair, and have emerged as potential effective vehicles for targeted drug delivery or direct therapeutic applications (Saravanan et al., 2023; Zheng et al., 2025).

**FIGURE 10:**
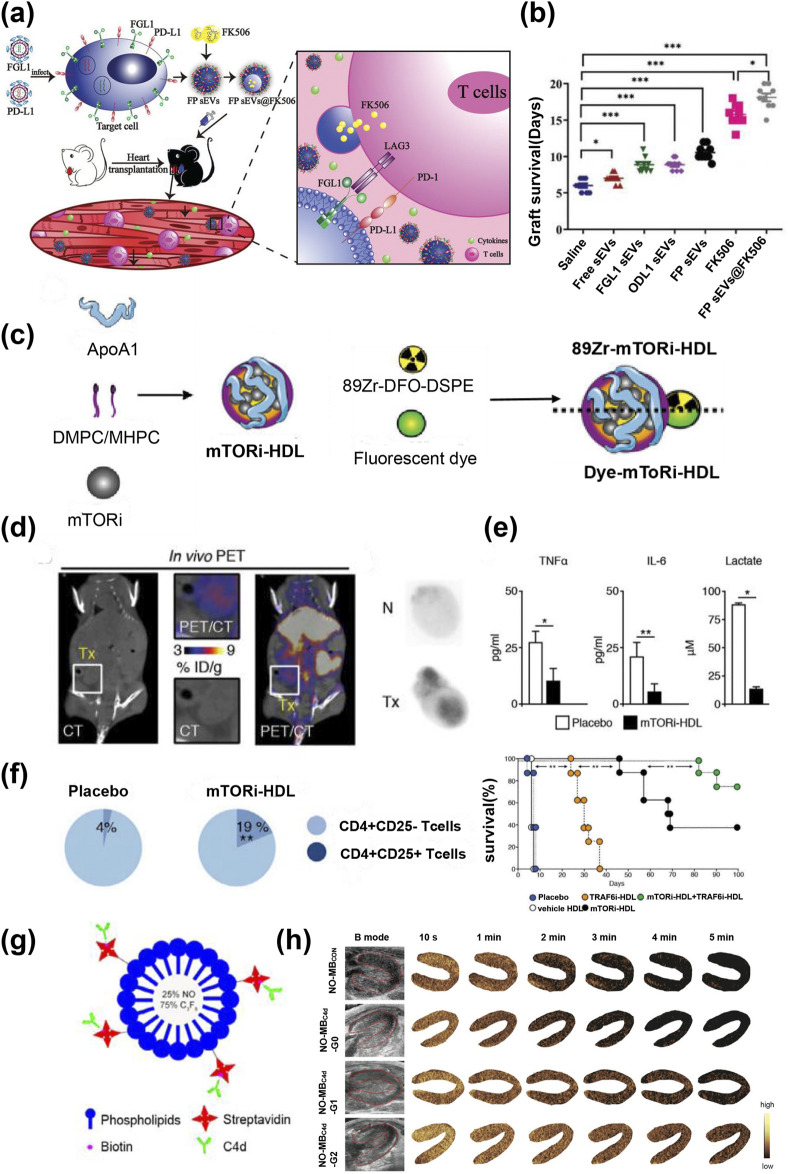
**(a)** A schematic model showing FP sEVs@FK506 suppress T-cell activation and, thus, inhibit cardiac allograft rejection. **(b)** Survival curves of cardiac allografts treated in different groups, n = 9, *p < 0.05, ***p < 0.001 (Tsai et al., 2022). **(c)** Preparation of mTORi-HDL labeled with either the radioisotope ^89^Zr or the fluorescent dyes DiO or DiR. **(d)** Micro-PET/CT 3D fusion image of heart transplanted mice after intravenous administration of ^89^Zr-mTORi-HDL; *ex vivo* autoradiography in native (N) and transplanted hearts (Tx). **(e)** Cytokine and lactate production of graft-infiltrating macrophages from either placebo or mTORi-HDL-treated recipients; n = 4, *p < 0.05; **p < 0.01. **(f)** Percentage of graft-infiltrating CD4^+^CD25^+^ Treg cells from placebo and mTORi-HDL-treated recipients; survival of grafts in each group, n = 7–8, **p < 0.01 (Braza et al., 2018). **(g)** Preparation scheme of NO-MB_C4d_. **(h)** Representative ultrasound images of myocardial regions at the indicated times after injection of MBs (red line in B-mode) (Liao et al., 2020).

Engineered EVs refers to the combination of EVs with other biomaterials or functional molecules through engineering technology. It aims to integrate the functional advantages of EVs with those of other materials to overcome the limitations of traditional EVs in applications, optimize therapeutic efficiency, and minimize side effects (Kumar et al., 2024). Cytotoxic T lymphocyte-associated antigen 4 (CTLA-4) is a leukocyte differentiation antigen that suppresses the immune response (Zhang H. et al., 2020). Programmed death receptor-1/programmed death ligand-1 (PD-1/PD-L1) signaling negatively regulates adaptive immune responses by inhibiting the activity of effector T cells and enhancing the function of immunosuppressive Tregs to prevent immune dysregulation (Lee et al., 2024). Xu et al. prepared macrophage membrane-derived extracellular nanovesicles that express both PD-L1 and CTLA-4, PD-L1/CTLA-4 that become intensively accumulated at the heart transplant site in the neck and inhibited T-cell activation and proliferation through a powerful dual immunosuppressive axis (Figures 9c,d) (Xu et al., 2020). Similarly, a research team used donor antigen on exosomes derived from donor immature dendritic cells (DEX) as “bait” to attract donor antigen-specific T cells, while leveraging the overexpressed PD-L1 on DEX as the killer “fish hook” to achieve specific immune regulation by targeting PD-1 on T cells (Luo et al., 2023). In addition, PD-L1 overexpressing nanocapsules can be used to encapsulate immunosuppressive drugs for combination therapy. Cai et al. used small vesicles of mesenchymal stem cells overexpressing FGL1/PD-L1 to encapsulate low-dose FK506 (sEVs@FK506) (Figure 10a), which function as an immune checkpoint by specifically binding to receptors LAG-3 and PD-1 on target cells (Tsai et al., 2022). The results revealed sEVs@FK506 inhibition of T-cell proliferation, decreased CD8^+^ T cell density and cytokine production in spleen and heart grafts, increased lymph node Tregs, and prolonged graft survival (Figure 10b). This combination therapy has further enhanced the effects of nanomedicine and has achieved good results in many clinical studies, thereby representing a promising direction for development.

With advances in research, scientists have developed hybrid EVs. Lu et al. developed a novel multifunctional fused extracellular nanovesicle (FNVs@RAPA) encapsulating rapamycin (RAPA) for treating IRI during the acute phase of HT and preventing AR. This system combines plant-derived EVs with mesenchymal stem cell membrane-derived nanovesicles (MSC), leveraging their ability to inhibit immune cell chemotaxis and suppress macrophage-mediated inflammation. Furthermore, the application of a ROS-responsive bioorthogonal chemistry approach promoted the accumulation of FNVs@RAPA at the cardiac graft site (Lu et al., 2024). CSF1/CD47 dual-targeting nanovesicles (NVs) derived from mesenchymal stem cells (iPSC-MSCs) alleviate immune rejection by synergistically inhibiting the phagocytic function and inflammatory response of inflammatory macrophages through blocking the CSF1/CSF1R and CD47/SIRPα pathways (Xu Z. et al., 2024). In addition to drugs, extracellular vesicles can also be used to load therapeutic genes. Xu et al. used siSLAMF6-loaded exosomes derived from endometrial regenerative cells to reduce the amount of sialic acid on CD4^+^ T cell surfaces, inhibit the activation of T cell receptor signaling pathways, and thereby prolong allograft survival (Xu Y. et al., 2024). The activation of T cells by donor dendritic cells (DCs) is the main cause of AR in heart transplantation. Exosomes of bone marrow mesenchymal stem cells (BMSC) overexpressing miR-540-3p can reduce the expression of immune activation markers CD74 and NF-κB p65 in DCs and T cells, significantly enhancing immune tolerance and prolonging the survival period of cardiac allografts (He et al., 2024). Furthermore, Wu’s research demonstrated that dendritic cell exosomes with knockdown of Mettl3 gene expression can prevent immune rejection in mouse cardiac allograft models (Wu H. et al., 2020). Although EVs offer numerous advantages, genetic modification of plants can be time-consuming. To achieve large-scale use, several issues must be addressed, including the purity of EVs, isolation methods, and interference with other cells.

### 4.5 Nanotheranostics

The continuous development of nanomaterials allow researchers to upgrade standalone diagnostic or therapeutic tools into integrated theranostic nanoplatforms that combine “detection, imaging, and targeted therapy.” These platforms should be capable of tracking the distribution, targeting efficiency, and therapeutic responses to nanomedicines in the body in real time to facilitate the adjustment of treatment plans and ensure personalized, monitorable precision medicine, potentially offering revolutionary approaches for managing reactions to rejection. Intelligent nanotheranostic carriers can be developed by incorporating specific therapeutic and imaging agents into nanoparticles, leveraging the inherent properties of the materials, or combining both approaches. Further conjugation with targeting molecules (such as small ligands, peptides, aptamers, or antibodies) enhances recognition and interaction with specific cellular and subcellular targets. Nanotheranostic agents can be designed for activation by endogenous or exogenous stimuli, and materials capable of specifically recognizing diseased tissues can be employed to facilitate continuous imaging or sustained drug release in response to pathological stimuli. Compared to molecular agents and conventional nanomedicines, the advantages include their ability to facilitate patient stratification, controlled activation, enhanced therapeutic efficacy, and effective prediction and monitoring of treatment outcomes.

Braza et al. developed high-density lipoprotein–lipid composite nanoparticles loaded with the mTOR inhibitor rapamycin (mTORi-HDL), which were simultaneously labeled with the zirconium-89 radiolabel (Figure 10c). The study found that ^89^Zr-mTORi-HDL exhibits strong affinity for bone marrow-derived cells and subsequently infiltrates the transplanted heart. In a mouse heart transplantation model, the radioactivity intensity in the transplanted heart reached 2.3 times that of the native heart (Figure 10d). In addition, it successfully inhibited aerobic glycolysis in macrophages and the production of inflammatory factors (IL-6 and TNF-α) (Figure 10e), prevented CD8^+^ T cell-mediated immune responses, promoted the expansion of tolerant CD4^+^ Tregs, thereby enhancing immune tolerance in heart transplantation and prolonging the survival time of allografts (Figure 10f) (Braza et al., 2018) In another study, nanoparticles carrying DGKR therapeutic genes (PEG-g-PEI/pDNA-DGKR) were functionalized with CD3 single-chain antibodies (scAbCD3) and superparamagnetic iron oxide nanoparticles (SPION) to enable MRI imaging and gene therapy that targeted T cells. The results indicated that MRI effectively detected the aggregation of T cells in the transplanted heart, and the immune response to the heart transplant was significantly suppressed (Guo et al., 2012).

C4d is the fragment that remains after complement activation and covalently binds to the surface of vascular endothelial cells; it is considered to be a highly specific single marker for diagnosing antibody-mediated rejection (Luk et al., 2015; Tower et al., 2017). However, the detection of C4d relies on endocardial biopsy. In addition, Nitric oxide (NO) has been shown to play a key role in the immune system, primarily in the form of anti-inflammatory, antithrombosis, and immunosuppressive effects (Bogdan, 2015; García-Ortiz and Serrador, 2018). Liao et al. conjugated C4d antibody to NO-laden microbubbles using streptavidin for the diagnosis and treatment of AR of heart transplants in rats (Figure 10g). The results revealed that the ultrasound signal intensity and the clearance time of NO-MBC4d in the myocardial region increased with the rising C4d grade, thereby facilitating a noninvasive diagnosis of rejection (Figure 10h). In addition, the NO-MBC4d injection after heart transplantation significantly reduced the thrombosis and infiltration of inflammatory cells, which led to the alleviation of rejection and prolonged survival of allogeneic grafts (Liao et al., 2020).

## 5 Future prospects and clinical challenges

Over the past decade, researchers have demonstrated the great potential of nanomedicines in preclinical research for various diseases. The number of related research papers, clinical trials, nanotherapeutic drugs, and nanoparticle-containing imaging agents approved by the U.S. FDA has continued to increase, and this trend is expected to persist. Today, nanomedicine are been applied in various innovative fields such as medical diagnostics, vaccine development, immunotherapy, gene delivery, and tissue engineering (Selmani et al., 2022). A growing number of emerging nanomaterials have been developed for use in medical consumables, such as hydrogels, nanoscale patches, and nanocomposite scaffolds, all of which hold significant value for application in heart transplantation research. Nanomaterials have evolved from initially leveraging the enhanced permeability and retention (EPR) effect for drug delivery to progressively facilitating targeted drug delivery, imaging diagnostics, and personalized therapy. Multifunctional nanomaterials can integrate multimodal imaging with targeted drug delivery capabilities, thereby enabling real-time, precise disease diagnosis and simultaneous treatment (Yang et al., 2023). They also allow for monitoring therapeutic efficacy during treatment and adjusting drug administration regimens as needed, facilitating personalized and precise diagnosis and therapy. In addition, an “intelligent” system that can sense the microenvironment of the lesion (such as the pH value, specific enzymes, and redox levels) and respond accordingly can facilitate precise spatiotemporal control of drug release to maximize therapeutic efficacy and reduce off-target toxicity to healthy tissues (Li et al., 2025). The development of next-generation nanomedicines for medical applications has shifted from merely pursuing drug delivery efficiency to constructing more complex and integrated multifunctional platform.

The potential of nanomedicines is immense, but their biosafety remains a key challenge for clinical applications. Since nanoscale materials exhibit new physicochemical properties, they may cause unconventional toxicity. For instance, nanoparticles are more easily taken up by cells, but the risks of their long-term retention in the body, potential immunogenicity, and possible accumulation in organs (such as the liver and spleen) have not been fully clarified (Feng et al., 2020). The rules governing their absorption, distribution, metabolism, and clearance differ from those of macroscopic materials, making it difficult to directly apply traditional toxicological methods and highlighting the need for systematic long-term toxicological research. The nanotoxicity issues that nanomaterials themselves might trigger—such as thrombus formation, inflammatory responses, oxidative stress, or DNA damage—require particular vigilance, especially for vulnerable populations, including cardiovascular disease patients. The FDA’s draft guidelines state that before nanomedicines can be used in human patients, exhaustive characterization of the physical and chemical properties of nanomaterials, as well as their pharmacological parameters, is necessary to ensure that the efficacy and safety results obtained in laboratories are valid and align with those obtained during clinical analysis (de Vlieger et al., 2019). To address safety concerns, a growing number of researchers are focusing on biodegradable or biosourced materials (such as proteins, enzymes, extracellular vesicles, etc.) for use as drug carriers, since the materials also exert therapeutic effects and can be safely cleared by the body after fulfilling their function (Zhao et al., 2021). Research into the biodegradability and safety of these nanomaterials will be a Frontier for future research.

Achieving large-scale, highly reproducible, and low-cost production of nanomedicines with complex compositions and intricate structures remains one of the biggest challenges for industrialization. Minor variations in their physicochemical properties (such as size, morphology, surface charge, and drug loading capacity) can significantly alter their *in vivo* fate and therapeutic efficacy, making it difficult to meet the requirements of good manufacturing practice (GMP) (Xu et al., 2023). Currently, there are no agreed-upon guidelines or standards for the production, characterization, and regulation of nanoparticles so standardizing safety and functional tests for clinical use is equally challenging. Furthermore, most nanomedicines must be administered intravenously, which poses patients-compliance challenges. The ideal nanomedicine should be oral, stable, easy to store, and have a long dosing interval and a low dosing frequency, etc. To accelerate the clinical translation of nanomedicines, future researchers should focus on reducing their formulation complexity and simplifying their administration. Furthermore, most current research remains limited to animal experiments or cellular-level studies. The interspecies biological differences between animal models and humans can lead to inaccurate predictions regarding naoparticles clearance, targeting, and immune responses of nanoparticles, thereby affecting the pharmacokinetics and therapeutic outcomes of nanomaterials and thus hindering their clinical transformation. Additionally, researchers have employed different animal models in their preclinical research, making it difficult to compare the various research results of different models.

## 6 Conclusion

This article presents a review of the progress of research on nanomaterials for the diagnosis and treatment of cardiac transplant rejection. Despite the challenges involved, previous researchers have demonstrated that nanomedicines have great potential as promising approaches for treating cardiovascular diseases. A comprehensive understanding of the pathogenesis of cardiac transplant rejection may reveal new biomarkers and therapeutic targets, providing new insights for further innovation. The development of the next-generation nanomedicines is a multidisciplinary systematic project that depends on the deep integration of materials science, biology, medicine, and engineering to create smarter, safer, and more effective nanoscale diagnostic and therapeutic tools. For instance, nanorobots capable of autonomous navigation and targeted drug delivery, or the integration of imaging techniques and artificial intelligence models to precisely track the distribution of nanocarriers and their interaction with cells, and establish predictive models for the physicochemical properties of nanomaterials and their biological effects and toxicity to accelerate clinical transformation (Adir et al., 2020; Yan et al., 2023). It is essential to further develop and optimize theranostic nanomaterials. For instance, materials with independent diagnostic and therapeutic modalities could be developed to ensure that the released imaging contrast agents and drugs do not affect each other. Moreover, integrated sensing units could be constructed to facilitate real-time monitoring of treatment indicators (such as pH, enzyme activity, and drug concentration) and automatically adjust therapeutic outputs based on feedback, thereby delivering precise, efficient, and personalized medical care. It should be noted that there are still relevant gaps and challenges regarding nanomedicine that need to be urgently addressed. For instance, at present, there are no unified and standardized toxicological and ecological testing methods, which makes it difficult to adapt them to the traditional drug approval process. In the future, relevant testing standards and guidelines should be formulated to support assessments of the risks and therapeutic effects of nanomedicines, and a unified database should be established to achieve global supervision.
